# Daily steps and all-cause mortality: a meta-analysis of 15 international cohorts

**DOI:** 10.1016/S2468-2667(21)00302-9

**Published:** 2022-03

**Authors:** Amanda E Paluch, Shivangi Bajpai, David R Bassett, Mercedes R Carnethon, Ulf Ekelund, Kelly R Evenson, Deborah A Galuska, Barbara J Jefferis, William E Kraus, I-Min Lee, Charles E Matthews, John D Omura, Alpa V Patel, Carl F Pieper, Erika Rees-Punia, Dhayana Dallmeier, Jochen Klenk, Peter H Whincup, Erin E Dooley, Kelley Pettee Gabriel, Priya Palta, Lisa A Pompeii, Ariel Chernofsky, Martin G Larson, Ramachandran S Vasan, Nicole Spartano, Marcel Ballin, Peter Nordström, Anna Nordström, Sigmund A Anderssen, Bjørge H Hansen, Jennifer A Cochrane, Terence Dwyer, Jing Wang, Luigi Ferrucci, Fangyu Liu, Jennifer Schrack, Jacek Urbanek, Pedro F Saint-Maurice, Naofumi Yamamoto, Yutaka Yoshitake, Robert L Newton, Shengping Yang, Eric J Shiroma, Janet E Fulton

**Affiliations:** Department of Kinesiology and Institute for Applied Life Sciences, University of Massachusetts Amherst, Amherst, MA, USA; Department of Kinesiology and Institute for Applied Life Sciences, University of Massachusetts Amherst, Amherst, MA, USA; Department Kinesiology, Recreation, and Sport Studies, University of Tennessee, Knoxville, TN, USA; Department of Preventive Medicine, Northwestern University Feinberg School of Medicine, Chicago, IL, USA; Department of Sport Medicine, Norwegian School of Sport Sciences, Norwegian Institute of Public Health, Oslo, Norway; Department of Chronic Diseases and Ageing, Norwegian Institute of Public Health, Oslo, Norway; Department of Epidemiology, Gillings School of Global Public Health, University of North Carolina Chapel Hill, Chapel Hill, NC, USA; Division of Nutrition, Physical Activity, and Obesity, National Center for Chronic Disease Prevention and Health Promotion, Centers for Disease Control and Prevention, Atlanta, GA, USA; Department of Primary Care and Population Health, UCL Medical School, London, UK; Molecular Physiology Institute and Division of Cardiology, Department of Medicine, Duke University, Durham, NC, USA; Brigham and Women’s Hospital, Harvard Medical School, Boston MA, USA; Department of Epidemiology, Harvard T H Chan School of Public Health, Boston, MA; Division of Cancer Epidemiology and Genetics, National Cancer Institute, Rockville, MD, USA; Division of Nutrition, Physical Activity, and Obesity, National Center for Chronic Disease Prevention and Health Promotion, Centers for Disease Control and Prevention, Atlanta, GA, USA; Department of Population Science, American Cancer Society, Atlanta, GA, USA; Department of Biostatistics and Bioinformatics, Duke University Medical Center, Durham, NC; Department of Population Science, American Cancer Society, Atlanta, GA, USA; Agaplesion Bethesda Clinic, Research Unit on Ageing, Ulm, Germany; Department of Epidemiology, Boston University School of Public Health, Boston, MA, USA; Institute of Epidemiology and Medical Biometry, Ulm University, Ulm, Germany; Department of Clinical Gerontology, Robert Bosch Hospital, Stuttgart, Germany; IB University of Applied Health and Social Sciences, Stuttgart, Germany; Population Health Research Institute, St George’s, University of London, London, UK; Department of Epidemiology, University of Alabama at Birmingham, Birmingham, AL, USA; Department of Epidemiology, University of Alabama at Birmingham, Birmingham, AL, USA; Departments of Medicine and Epidemiology, Columbia University Irving Medical Center, New York, NY, USA; Department of Pediatrics, Center for Epidemiology and Population Health, Baylor College of Medicine, Houston, TX, USA; Department of Biostatistics, Boston University School of Public Health, Boston, MA, USA; Department of Biostatistics, Boston University School of Public Health, Boston, MA, USA; Department of Epidemiology, Boston University School of Public Health, Boston, MA, USA; Department of Medicine, Boston University School of Medicine, Boston, MA, USA; Department of Endocrinology, Diabetes, Nutrition and Weight Management, Boston University School of Medicine, Boston, MA, USA; Department of Community Medicine and Rehabilitation, Unit of Geriatric Medicine, Umeå University, Umeå, Sweden; Department of Public Health and Clinical Medicine, Section of Sustainable Health, Umeå University, Umeå, Sweden; Department of Community Medicine and Rehabilitation, Unit of Geriatric Medicine, Umeå University, Umeå, Sweden; Department of Public Health and Clinical Medicine, Section of Sustainable Health, Umeå University, Umeå, Sweden; School of Sport Sciences, UiT The Arctic University of Norway, Tromsø, Norway; Department of Sport Medicine, Norwegian School of Sport Sciences, Norwegian Institute of Public Health, Oslo, Norway; Department of Sport Medicine, Norwegian School of Sport Sciences, Norwegian Institute of Public Health, Oslo, Norway; Department of Sport Science and Physical Education, University of Agder, Norway; Menzies Institute for Medical Research, University of Tasmania, Hobart, TAS, Australia; Menzies Institute for Medical Research, University of Tasmania, Hobart, TAS, Australia; Nuffield Department of Women’s and Reproductive Health, University of Oxford, Oxford, UK; Murdoch Children’s Research Institute, Melbourne, VIC, Australia; Murdoch Children’s Research Institute, Melbourne, VIC, Australia; Intramural Research Program, National Institute on Aging, Baltimore, MD, USA; Department of Epidemiology, Johns Hopkins School of Medicine, Baltimore, MD, USA; Department of Epidemiology, Johns Hopkins School of Medicine, Baltimore, MD, USA; Center on Aging and Health, Johns Hopkins School of Medicine, Baltimore, MD, USA; Center on Aging and Health, Johns Hopkins School of Medicine, Baltimore, MD, USA; Division of Geriatric Medicine and Gerontology, Department of Medicine, Johns Hopkins School of Medicine, Baltimore, MD, USA; Division of Cancer Epidemiology and Genetics, National Cancer Institute, Rockville, MD, USA; Faculty of Collaborative Regional Innovation, Ehime University, Matsuyama, Ehime, Japan; Institute for Pacific Rim Studies, Meio University, Nago, Okinawa, Japan; Pennington Biomedical Research Center, Baton Rouge, LA, USA; Pennington Biomedical Research Center, Baton Rouge, LA, USA; Laboratory of Epidemiology and Population Sciences, National Institute on Aging, Baltimore, MD, USA; Division of Nutrition, Physical Activity, and Obesity, National Center for Chronic Disease Prevention and Health Promotion, Centers for Disease Control and Prevention, Atlanta, GA, USA

## Abstract

**Background:**

Although 10 000 steps per day is widely promoted to have health benefits, there is little evidence to support this recommendation. We aimed to determine the association between number of steps per day and stepping rate with all-cause mortality.

**Methods:**

In this meta-analysis, we identified studies investigating the effect of daily step count on all-cause mortality in adults (aged ≥18 years), via a previously published systematic review and expert knowledge of the field. We asked participating study investigators to process their participant-level data following a standardised protocol. The primary outcome was all-cause mortality collected from death certificates and country registries. We analysed the dose–response association of steps per day and stepping rate with all-cause mortality. We did Cox proportional hazards regression analyses using study-specific quartiles of steps per day and calculated hazard ratios (HRs) with inverse-variance weighted random effects models.

**Findings:**

We identified 15 studies, of which seven were published and eight were unpublished, with study start dates between 1999 and 2018. The total sample included 47 471 adults, among whom there were 3013 deaths (10.1 per 1000 participant-years) over a median follow-up of 7.1 years ([IQR 4.3–9.9]; total sum of follow-up across studies was 297 837 person-years). Quartile median steps per day were 3553 for quartile 1, 5801 for quartile 2, 7842 for quartile 3, and 10 901 for quartile 4. Compared with the lowest quartile, the adjusted HR for all-cause mortality was 0.60 (95% CI 0.51–0.71) for quartile 2, 0.55 (0.49–0.62) for quartile 3, and 0.47 (0.39–0.57) for quartile 4. Restricted cubic splines showed progressively decreasing risk of mortality among adults aged 60 years and older with increasing number of steps per day until 6000–8000 steps per day and among adults younger than 60 years until 8000–10 000 steps per day. Adjusting for number of steps per day, comparing quartile 1 with quartile 4, the association between higher stepping rates and mortality was attenuated but remained significant for a peak of 30 min (HR 0.67 [95% CI 0.56–0.83]) and a peak of 60 min (0.67 [0.50–0.90]), but not significant for time (min per day) spent walking at 40 steps per min or faster (1.12 [0.96–1.32]) and 100 steps per min or faster (0.86 [0.58–1.28]).

**Interpretation:**

Taking more steps per day was associated with a progressively lower risk of all-cause mortality, up to a level that varied by age. The findings from this meta-analysis can be used to inform step guidelines for public health promotion of physical activity.

## Introduction

Physical activity can reduce morbidity and mortality due to multiple chronic conditions, including cardiovascular disease, type 2 diabetes, and several cancers, and is associated with better quality of life.^1,2^ The number of steps acquired per day is a simple measure of physical activity. Monitoring daily steps is more feasible than ever for the general public as fitness trackers and mobile devices have become increasingly popular.^3,4^ Although the goal of 10 000 steps per day is widely promoted as being optimal for general health, it is not based on evidence, but instead originates from a marketing campaign in Japan.^5^ Expert committees from the WHO 2020 Physical Activity Guidelines and US 2018 Physical Activity Guidelines identified a gap in research on the dose–response association between volume and intensity of physical activity and health outcomes, including physical activity measured by step volume and rate.^1,2^

The optimal number of steps needed to reduce the risk of mortality might be affected by characteristics such as age or sex. Walking volume and pace decrease with age and might differ by sex; hence, the distribution of steps differs in younger and older adults and by sex.^6,7^ Findings from large prospective studies have shown mortality risk levels off for older women (aged ≥62 years) at 7500 steps per day^5^ and among a nationally representative sample of US and Norwegian adults (aged ≥40 years) at approximately 8000–12 000 steps per day.^6^ Several observational studies have shown stepping rate, a marker of intensity, is inversely associated with mortality; however, when adjusted for volume of steps per day, step rate was no longer associated with mortality.^5,6,8^ A meta-analysis observed a linear association between step volume and mortality from seven studies, observing large heterogeneity among studies and did not report associations by age, sex, or stepping rate.^9^

Here, we aimed to complete a meta-analysis on steps per day and mortality, addressing the limitations of previous studies. We aimed to include a larger sample of studies than previous meta-analyses and to collect data across age groups and by sex to generate robust evidence to inform a daily step count guideline. Our primary objective was to assess the dose–response association between steps per day and all-cause mortality and determine whether this association varied by age and sex. A secondary objective was to assess the association between stepping rate and all-cause mortality. We hypothesised that a dose–response association exists between steps per day and mortality and that the association would differ between younger and older adults.

## Methods

### Search strategy and selection criteria

This meta-analysis was completed in association with The Steps for Health Collaborative, which is an international consortium that was formed to determine the association between device-measured volume and rate of steps and prospective health outcomes among adults.

Two strategies were used to identify studies for this meta-analysis. First, we identified studies through a systematic review of daily step count and associations with all-cause mortality, cardiovascular disease, and dysglycaemia, the findings of which have been published previously.^10^ Briefly, we searched MEDLINE, Embase, CINAHL, and Cochrane Library databases for publications in English from database inception to Aug 1, 2019. Search terms were related to daily step count measured by pedometer or accelerometer and to mortality, cardiovascular disease, and dysglycaemia. Eligibility criteria included longitudinal design, adult participants (aged ≥18 years), and non-patient populations, and that the study reported an association between daily step counts and mortality. The previous systematic review was registered with PROSPERO (CRD42020142656).^10^ Five studies were identified through this systematic review, a number that was deemed too few for a meta-analysis. Therefore, we used a second strategy to identify additional studies for the current meta-analysis.

Additional studies were identified through Collaborative members’ awareness of ongoing and unpublished studies measuring steps and mortality. These studies were also required to meet the inclusion criteria stipulated in the previous systematic review. The investigators of studies found to be eligible were approached by AEP to ask whether they would participate in this meta-analysis.

We used the Newcastle Ottawa quality assessment scale to assess the methodological quality of each study.^11^ Risk of bias assessments were done independently by two reviewers (AEP and SB), and disagreements were resolved by consensus between the two reviewers.

### Individual study-level data processing

We asked the investigators of participating studies to process their participant-level data according to a standardised protocol developed by The Steps for Health Collaborative to limit heterogeneity in our analyses across studies (appendix pp 34–60). In each study, participants wore a step counting device for 1 week, considered baseline in this study, and then were followed up for death from any cause. Investigators were asked to quantify step volume as steps per day, averaged over all days for which step data were collected. Studies that quantified stepping rate used one or more of four measures reported in previous studies on steps and mortality.^5,6,8^ We asked the investigators of each study to calculate peak 30 min and 60 min stepping rates as the highest number of steps accumulated over 30 min and 60 min periods (not necessarily consecutively) throughout each day and as a mean over all days. We also asked study investigators to calculate stepping rate as the time (in min) spent walking at 40 steps per min or faster (defined as intentional walking) and 100 steps per min or faster (defined as a moderate rate walking pace).^12^ Our primary outcome was all-cause mortality collected from death certificates and country registries.

### Individual study-level analyses

The Steps for Health Collaborative established a standardised analytical plan for study investigators to complete. Investigators of participating studies were asked to categorise step volume into quartiles across the study population and examine associations with all-cause mortality (referenced against the lowest quartile) using Cox proportional hazards regression (satisfying proportional hazards assumptions) producing hazard ratios (HRs) and 95% CIs. Investigators of participating studies completed models for each study’s overall sample, by age group and by sex where applicable. Age was grouped into younger (<60 years) and older (≥60 years) groups on the basis of WHO’s definition of older people from the 2020 Decade of Healthy Ageing Baseline Report.^13^ Investigators of participating studies constructed two models: model 1 adjusted for age and sex and model 2, the final model, adjusted for sociodemographic factors, lifestyle behaviours, and health indicators that are known to affect the association between steps per day and all-cause mortality. Model 2 also adjusted for age, sex, race and ethnicity, education or income, body-mass index, and study-specific covariates for chronic disease (eg, diabetes, blood pressure, history of cardiovascular disease or cancer, and medications), self-rated health or functional status, accelerometer wear time, and lifestyle factors (eg, smoking and alcohol; appendix p 5). Investigators of participating studies were asked to complete sensitivity analysis excluding deaths within the first 2 years of follow-up.

For studies with stepping rate measures, we used the same analytical approach for model 1 and model 2. Model 3 adjusted for all covariates from model 2 plus steps per day using the residual method in which stepping rate was regressed on steps per day and the resulting stepping rate residuals and steps per day were independent variables in the model.^5,14^

### Data analysis

We summed the total number of participants, deaths, and person-years of follow-up across all studies. For the total sample, we calculated median (IQR) steps per day by quartile from the medians of each individual study. We calculated risk differences and 95% CIs as comparison quartile minus reference quartile (ie, the quartile with the lowest number of steps per day). We assessed differences in median steps per day using the Wilcoxon rank-sum test. We meta-analysed effect estimates using inverse-variance weighted random-effects models, calculating pooled HRs and 95% CIs. The final adjusted model (model 2) was the primary model. Because of the known associations of age and sex with physical activity,^1^ we did a priori stratified analyses by age and sex for the associations between mortality and steps per day. We calculated *I*^2^ heterogeneity values, which were considered to be low (<25%), moderate (25–75%), or high (>75%).^15^ We assessed presence of study bias using funnel plots comparing study HRs against SEs and Egger’s test for funnel plot symmetry.^16^

We used log-transformed HRs from model 2 to generate restricted cubic spline models using knots at the 25th, 50th, and 75th percentiles of total steps per day.^17^ We used the Wald test to test for non-linearity by examining the null hypothesis that the regression coefficient of the spline transformation was equal to zero.^18^ We examined model fit using de-correlated residuals versus exposure plots and the coefficient of determination.^18^ We assessed age (aged <60 years *vs* ≥60 years) and sex subgroup differences in curves using multiplicative interaction terms. We excluded one study^19^ from all spline analyses because step data were processed with a low frequency extension filter, which significantly inflates steps per day.^20^

We also did a series of sensitivity analyses. We investigated the potential for reverse causation by excluding participants at the study level who died within the first 2 years of follow-up. We stratified studies by average length of follow-up and compared those with less than 6 years of follow-up and 6 years or longer of follow-up.^21^ We compared studies stratified by publication status (published *vs* unpublished). We did an analysis using the leave-one-out approach, excluding one study at a time, to ensure that the results were not simply due to one large study or a study with an extreme result. Furthermore, we used a leave one-device-out approach, in which we excluded all studies that used a specific step-monitoring device, to determine if the dose–response estimates of steps were affected by any single device. We also reanalysed our data using a fixed-effects inverse-variance method.

p values of less than 0.05 were considered to be statistically significant. We did meta-analyses using R (version 4.0) and SAS (version 9.4).

### Role of the funding source

The staff of the funder had no role in data collection or data analysis, but did have a role in the study design, data interpretation, and writing of the report.

## Results

**Figure 1:**
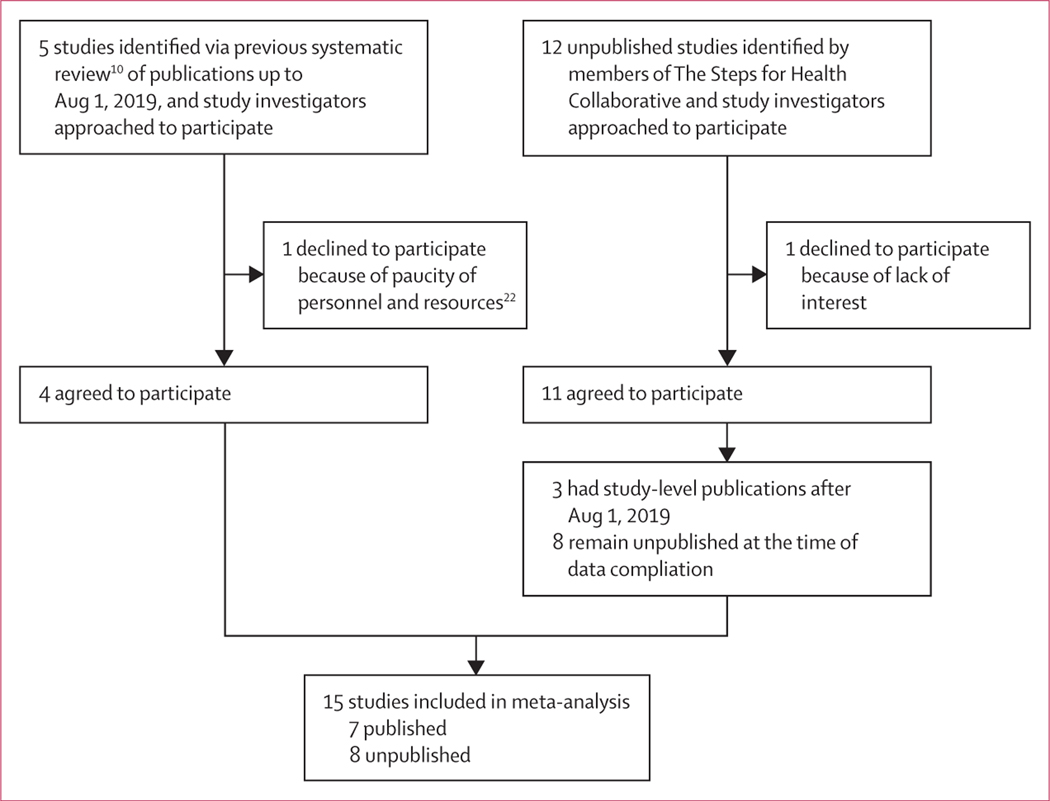
Study selection

We identified 15 studies that were eligible for inclusion in our meta-analysis (figure 1), including four studies in Europe, one in Japan, one in Australia, eight in the USA, and one that included data from 40 countries (table; appendix pp 3–4). Seven studies were published^5,6,8,17,23,24,26^ and eight were unpublished at the time of data compilation,^19,27–33^ with study start dates ranging between 1999 and 2018.

The total sample included 47 471 participants (individual-level mean age 65.0 years [SD 12.4], 32 226 [68%] were female, and >70% were of White race [appendix pp 6–8]), with a median study follow-up time of 7.1 years (range 2.7–13.5 [IQR 4.3–9.9]; total sum of follow-up across studies was 297 837 person-years). The overall median of the median steps per day was 6495 [IQR 4273–8768]. Adults younger than 60 years had significantly higher median steps per day (7803 [IQR 5377–10 352]) than did adults aged 60 years and older (5649 [IQR 3686–8092]; p=0.033). A total of 3013 deaths were reported (10.1 per 1000 participant-years). The Newcastle Ottawa quality scores were high, ranging from 7 to 9 out of a possible 9 points (appendix p 10).

**Figure 2:**
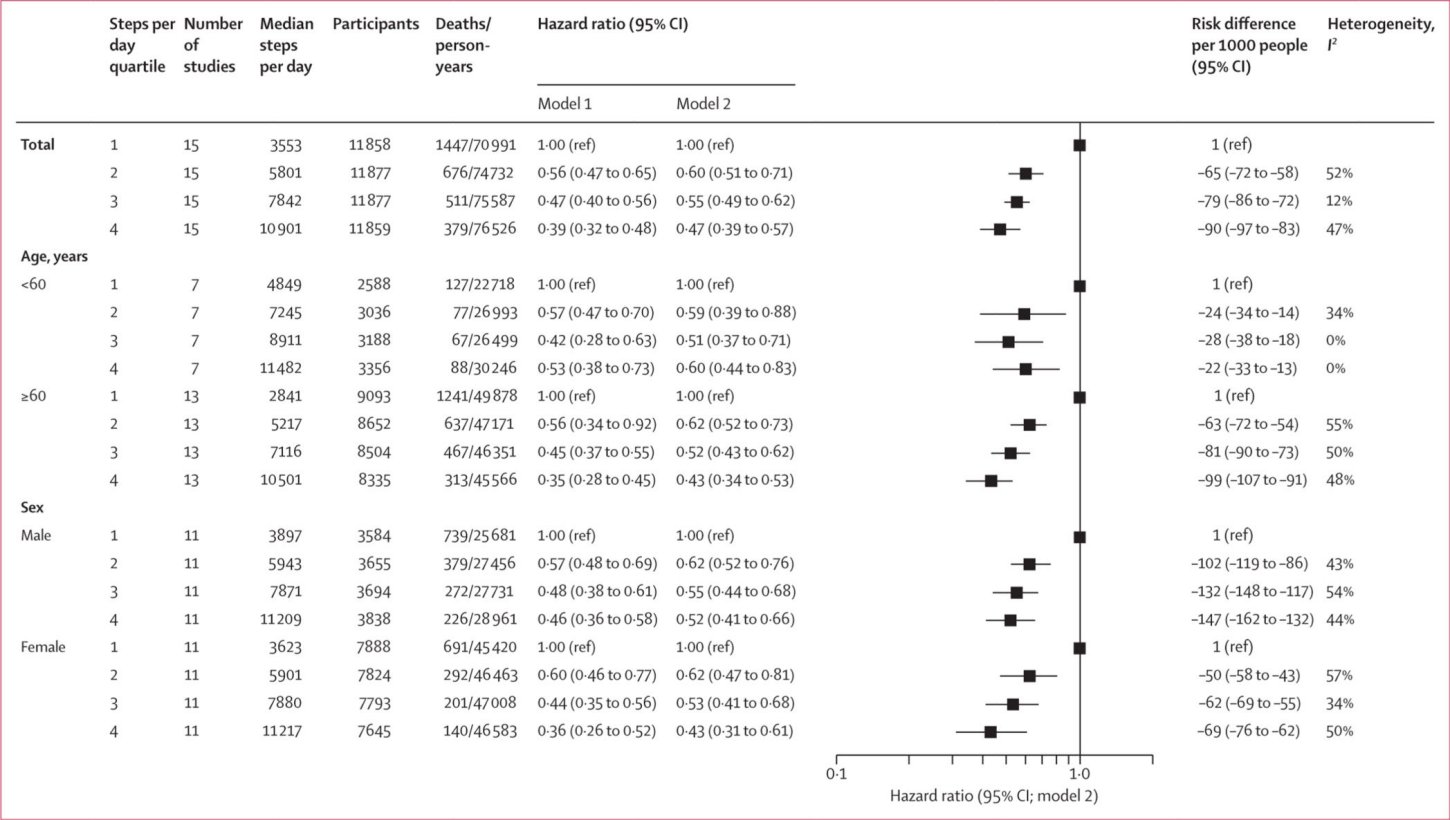
Association between steps per day and all-cause mortality, in all participants, and by age and sex Model 1 adjusted for age and sex (if applicable). Model 2 was further adjusted for device wear time, race and ethnicity (if applicable), education or income, body-mass index, plus study-specific variables for lifestyle, chronic conditions or risk factors, and general health status. The x-axis of the plot is on the log scale.

Compared with the lowest quartile of steps per day, higher quartiles of steps per day were associated with a reduced risk of mortality in the overall sample (figure 2; appendix p 13). Funnel plots had minor asymmetry for the second and third quartile comparisons among lower weighted studies with visual inspection (appendix p 14). Egger’s test for symmetry suggested no evidence of study selection bias (appendix p 14). There was a non-linear, dose–response association between steps per day and all-cause mortality in the spline model (p_non-linearity_<0.0001). The lowest HR was observed at approximately 7000–9000 steps per day in the overall sample (appendix p 15).

**Figure 3:**
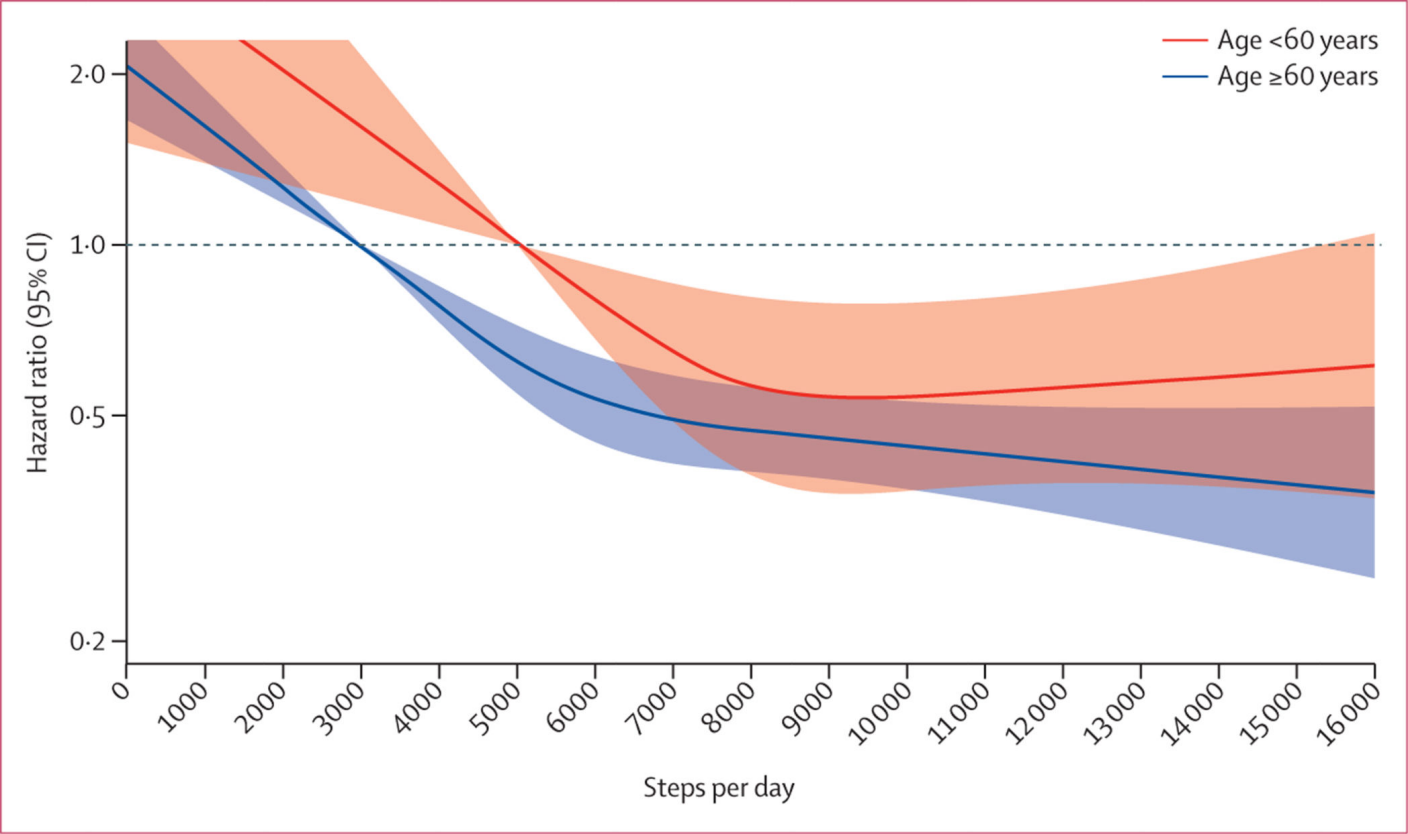
Dose-response association between steps per day and all-cause mortality, by age group Thick lines indicate hazard ratio estimates, with shaded areas showing 95% CIs. Reference set at the median of the medians in the lowest quartile group (age ≥60 years = 3000 steps per day and <60 years = 5000 steps per day). Model is adjusted for age, accelerometer wear time, race and ethnicity (if applicable), sex (if applicable), education or income, body-mass index, and study-specific variables for lifestyle, chronic conditions or risk factors, and general health status. p_interaction_=0.012 by age group. 14 studies included in spline analysis, excluded Baltimore Longitudinal Study of Aging.^19^ The y-axis is on a log scale.

HRs for risk of mortality by age group (<60 years and ≥60 years) are shown in figure 2 and the appendix (pp 16–17). There was a significant interaction (p=0.012) by age group in the spline model (figure 3). The number of daily steps at which the HR for mortality plateaus among adults aged 60 years and older was approximately 6000–8000 steps per day and among adults younger than 60 years was approximately 8000–10 000 steps per day (figure 3).

The HRs for mortality were similar for females and males (figure 2; appendix pp 20–21). The interaction by sex in the spline model was not significant (p=0.11). For males and females, the lowest HR for mortality was seen at approximately 7000–9000 steps per day (appendix p 23).

**Figure 4:**
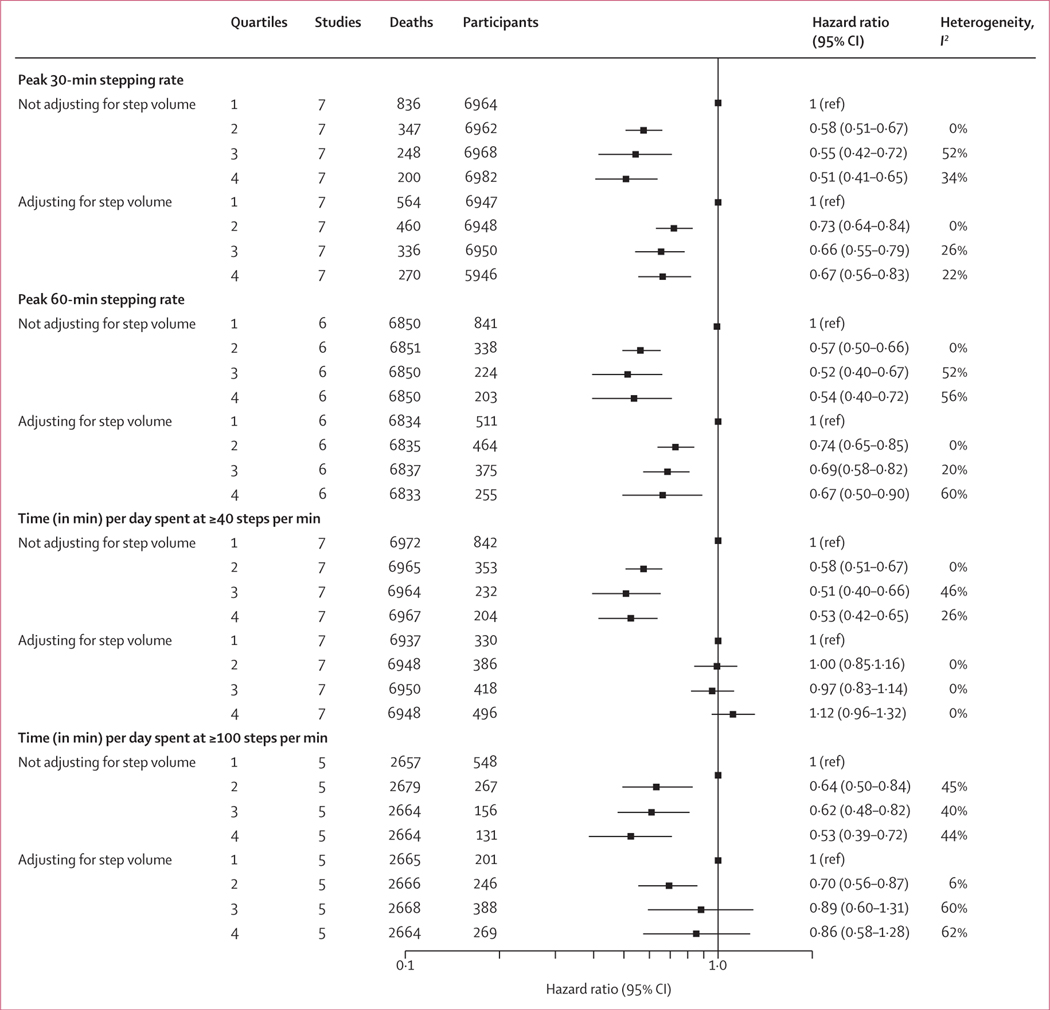
Association between stepping rate with all-cause mortality, with and without adjustment for total step volume Hazard ratios and 95% CIs are adjusted for age, device wear time, race and ethnicity (if applicable), sex (if applicable), education or income, body-mass index, and study-specific variables for lifestyle, chronic conditions or risk factors, and general health status. The model with additional adjustment for step volume uses the residual method for the rate variable. The x-axis is on a log scale.

Seven studies reported stepping rate measures (table). Median peak 30-min stepping rate was 64.1 steps per min (IQR 52.9–80.5) and 60-min stepping rate was 57.5 steps per min (46.2–70.9). Median time spent walking at a rate of 40 steps per min or faster was 51.4 min (23.3–87.4) and at 100 steps per min or faster was 5.2 min (1.3–15.2). Higher stepping rates were associated with lower risk of mortality without adjustment for total steps (model 2; figure 4). The association between peak 30-min and peak 60-min rate measures and mortality remained significant after adjusting for steps per day (appendix pp 24–25). After adjusting for step volume, time spent walking at 40 steps per min or faster and at 100 steps per min or faster were not associated with mortality, except for the first versus second quartiles at a rate of 100 steps per min or faster (figure 4; appendix pp 26–27).

Sensitivity analyses excluding deaths within the first 2 years of follow-up showed the association between steps per day quartiles and mortality was attenuated but remained significant (appendix pp 28–29). The association between step counts and mortality was stronger in the six studies with fewer than 6 years of follow-up (HR 0.32 [95% CI 0.25–0.41]) than among the nine studies with 6 years of follow-up or more (0.57 [0.49–0.66]) when comparing the lowest and highest quartile (appendix p 30). There was a significantly lower HR for published (0.54 [0.42–0.68]) than unpublished studies (0.73 [0.63–0.85]) when comparing the first and second quartile (appendix p 31). We found no appreciable differences in the association between steps per day and mortality when excluding any one study or step-counting device (appendix p 33). When reanalysing the data using a fixed-effects inverse-variance method, we found no change in the results (appendix p 12). In main analyses, heterogeneity (*I*^2^) was low to moderate, ranging from 0 to 57% across quartiles (figure 2).

## Discussion

In this meta-analysis of 15 studies, seven published and eight unpublished, we found that taking more steps per day was associated with progressively lower mortality risk, with the risk plateauing for older adults (aged ≥60 years) at approximately 6000–8000 steps per day and for younger adults (aged <60 years) at approximately 8000–10 000 steps per day. We found inconsistent evidence that step intensity had an association with mortality beyond total volume of steps.

Our findings add to the body of research on steps and health by describing a curvilinear association and range in steps per day associated with all-cause mortality. The curvilinear association and 50–60% lower risk in the higher steps per day quartiles than in the lowest steps per day quartile is similar to the association and risks observed for time spent doing moderate-to-vigorous intensity physical activity and mortality,^17^ and study-level publications on steps and mortality.^5,6,8,25^ The steep early slope of the dose–response curve suggests increasing steps might be beneficial in terms of reducing risk of mortality, particularly among individuals who have lower step volumes. We observed a plateau in risk reduction, which varied by age group. We did not find that high step volumes were associated with increased risk of mortality.^34^ Furthermore, in sensitivity analyses, we found stronger associations among studies with shorter follow-up than in those with longer follow-up,^21^ suggesting that more recent physical activity might be more important for associations with mortality.

Contrary to the curvilinear dose response observed in our analysis, a recent steps and mortality meta-analysis of seven studies found a linear association for 2700–17 500 steps per day; however, this study was limited by sparse data being available at the upper end of the steps distribution, with only three effect estimates provided above 12 500 steps per day.^9^ Because of the small number of studies included, this meta-analysis was unable to provide robust subgroup analyses and, therefore, was unable to examine associations by age or sex. Here, we included 15 studies and applied a standardised, meta-analytical method for data synthesis across studies, strengthening the reliability of our findings.

We found that thresholds of steps per day were different for younger and older adults because the steps per day versus mortality spline curves varied by age group. The curvilinear shape of the step count to mortality association was similar for older and younger adults, but the step volume associated with a given HR differed by age. In a study of older women (aged ≥62 years) by Lee and colleagues,^5^ the mortality risk plateaued at 7500 steps per day.^5^ We observed a similar plateauing at 6000–8000 steps per day for older individuals, and included both sexes and a slightly wider age group to enable us to identify ranges of steps per day for younger and older age groups, and by sex. As age increases, mobility limitations, decreases in aerobic capacity, and biomechanical inefficiencies might restrict the possible number of steps per day older adults can accumulate.^35,36^ The association between daily steps and all-cause mortality might start at lower step volumes for older adults because of lower absolute step volume for the same relative step intensity and physiological stimulus than for younger adults. Therefore, older adults might require a lower number of steps to gain similar improvements in health benefits.^37^

We found an association between stepping rate (cadence) and all-cause mortality with some, but not all, rate measures.^5,6^ Increasing daily peak stepping rate in any (not necessarily consecutive) 30 min or 60 min period, independent of steps per day, was associated with reduced mortality.^12^ Conversely, adjusting for step volume, time spent walking at 40 steps per min or faster and 100 steps per min or faster were not associated with mortality. Peak stepping rate might better reflect fitness levels than thresholds of time spent walking at 40 or 100 steps per min or faster, and fitness is a strong predictor of mortality,^38^ which might partially explain why peak stepping rate might be more strongly related to mortality than the 40 and 100 steps per min thresholds. The time threshold measures we used here were developed in laboratory settings^12,39^ and might not represent real-world patterns of walking. Peak stepping rate variables were more normally distributed than thresholds measures, allowing for easier detection of differences.^12^ For example, most participants spent little time walking at 100 steps per min or faster (median 5.2 min per day [IQR 1.3–15.2]). Time spent walking at a speed slower than 100 steps per min might be considered for future observational studies of the association between walking with health outcomes. Disentangling the health associations of stepping rates from step volume in daily life is difficult because individuals who walk at a faster pace usually accumulate more steps per day than those who walk at a slower pace. Trials prescribing different stepping rate groups while maintaining the same total step volume might be needed to fully examine the association between stepping rate and intermediate health outcomes (eg, hypertension or diabetes).^1^ Taken together, our findings were inconclusive when determining if step intensity has additional mortality benefits beyond that associated with total steps.

The implications of our findings extend to health care and public health. Steps per day is a simple and easy to interpret measure that can enhance clinician–patient and public health communication for monitoring and promoting physical activity. Wearable devices that monitor steps, such as smartphones and fitness trackers, have substantially increased in popularity over the past decade and this popularity is expected to continue to increase.^3,4^ Many consumers rely on the number of steps provided from these devices to monitor their physical activity.

Our study has several limitations. The data are derived from observational studies; therefore, causal inferences cannot be made. We focused on all-cause mortality; however, the associations between steps and other health outcomes are important considerations when developing guidelines or providing clinical advice. Although we attempted to control for sociodemographic, lifestyle, and health status factors in our analyses, residual confounding and reverse causality might still be present. Steps were measured at a single timepoint. 1 week of device-measured steps has relative stability over several years,^40^ but does not account for changes in steps per day over time. In this meta-analysis we used study-level data, and although we standardised our analyses across studies, heterogeneity in participants between studies (eg, demographics, health status) and design (eg, step-counting device, covariates) might not be fully accounted for compared with in individual-level pooled meta-analyses. We selected prespecified knots in splines, which risks model misspecification. All included studies were in high-income countries and participants were volunteers primarily among White populations, restricting generalisability of the findings. Future research should emphasise monitoring and promoting steps in populations at higher risk of mortality (eg, some race and ethnicity groups, low socioeconomic status, and individuals with or without high risk for chronic diseases). Since the development of this meta-analysis collaboration, to our knowledge, two studies on steps and mortality^41,42^ have been published. The findings of these two studies, which included primarily older adults, are consistent with our results, with a greater number of daily steps being significantly associated with a decreased risk of all-cause mortality.

Device type, wear location, and walking speed and duration can affect the accuracy of step estimates. Step counts obtained from research and consumer devices are highly correlated but can vary by 20% or more;^20^ therefore, estimates of steps per day reported here might not precisely match all devices. Stepping rate was measured as the number of steps accumulated per min rather than the number of steps while in motion and, therefore, might not adequately capture short walking periods, which are common in daily life.^43^ Additionally, some devices might not detect all steps at very slow walking speeds.^44^ Therefore, devices might underestimate steps particularly among frail older adults. Most of the participating studies used devices worn at the hip, whereas many consumer devices are worn on the wrist and can provide different estimates.^20^

This meta-analysis has several strengths. The participant population was geographically diverse, and so the associations were generated with greater precision and relevance to a diverse population of individuals worldwide than would be possible in individual, country-level studies. Use of measures recorded by devices such as step counters and accelerometers might more accurately reflect the strength of the association between movement and mortality than self-reported activity.^45^ Each study used a consistent methodological approach to minimise heterogeneity. Unpublished studies were invited to participate, which would have reduced publication bias. Positive findings tend to be published earlier and more often than negative findings;^46^ therefore, if we had only relied on published evidence the estimated pooled effect size might have been overestimated. We found associations between daily steps and all-cause mortality in both published and unpublished studies, providing robust evidence for this association.

There are currently no evidence-based public health guidelines recommending the number of steps per day for health benefits. Our findings suggest mortality benefits, particularly for older adults, can be seen at levels less than the popular reference of 10 000 steps per day. Adults taking more steps per day have a progressively lower risk of all-cause mortality, up to a level that varies by age. Our findings can be used to inform step guidelines for clinical and population promotion of physical activity.

## Supplementary Material

MMC1

## Figures and Tables

**Table:** Selected characteristics of included studies

	Publication	Country	Study entry	Step-monitoring device (wear location)	Stepping rate measures available	Participants	Mean age, years (SD)	Female participants	Mean follow-up, years	Deaths during follow-up

**Published**										
British Regional Heart Study (BRHS)	Jefferis et al (12019)^23^	UK	2010–12	ActiGraph GT3X (waist)	None	1397	78.4 (4.6)	0	4.7	240
Coronary Artery Risk Development in Young Adults (CARDIA)	Paluch at al (2021)^8^	USA	2005–06	ActiGraph 7164 (waist)	Peak 30 min, peak 60 min, time at ≥40 steps per min, time at ≥100 steps per min	2110	45.2 (3.6)	1203 (57%)	10.2	72
National Health and Nutrition Examination Survey (NHANES)	Saint Maurice et al (2020)^6^	USA	2005–06	ActiGraph 7164 (waist)	Peak 30 min, peak 60 min, time at ≥40 steps per min, time at ≥100 steps per min	2382	60.1 (13.3)*	1189 (50%)	10.0	507
Niigata Elderly Study (NES)	Yamamoto et al (2018)^24^	Japan	1999	EC-100S, YAMASA (waist)	None	416	71 (0)	189 (45%)	9.8	76
Norwegian National Physical Activity Surveillance 1 (NNPAS1)	Hansen et al (2020)^25^	Norway	2008–09	ActiGraph GT1M (waist)	None	3043	49.9 (14.9)	1627 (53%)	8.9	122
Tasped Pooled Cohort Study (Tasped)	Dwyer et al (2015)^26^	Australia	2000	Yamax SW-200 and Omrom-HJ-003 and Omron HJ-102 (waist)	None	2576	58.7 (13.2)	1350 (52%)	11.1	219
Women’s Health Study (WHS)	Lee et al (2019)^5^	USA	2011	ActiGraph GT3X (waist)	Peak 30 min, peak 60 min, time at ≥40 steps per min	16 741	72.0 (5.7)	16 741 (100%)	4.3	504
**Unpublished**										
Activity and Function in the Elderly in Ulm (ActiFE)	NA	Germany	2009–10	activPAL (thigh)	Peak 30 min, peak 60 min, time at ≥40 steps per min, time at ≥100 steps per min	1240	75.4 (6.5)	712 (57%)	8.2	367
Atherosclerosis Risk in Communities Study (ARIC)	NA	USA	2016–17	ActiGraph GT3X (waist)	Peak 30 min, time at ≥40 steps per min	452	78.4 (4.7)	266 (59%)	2.9	25
Baltimore Longitudinal Study of Aging (BLSA)	NA	USA	2016	ActiGraph GT3X-LFE (wrist)	Peak 30 min, peak 60 min, time at ≥40 steps per min, time at ≥100 steps per min	382	76.1 (8.9)	201 (53%)	2.7	22
Cancer Prevention Study-3 (CPS-3)	NA	USA	2015	ActiGraph GT3X (waist)	None	720	52.7 (10.0)	428 (59%)	3.5	6
Framingham Heart Study (FHS)	NA	USA	2008–14	Actical (model number 198–0200-00; waist)	Peak 30 min, peak 60 min, time at ≥40 steps per min, time at ≥100 steps per min	4548	55.3 (13.9)	2444 (54%)	7.1	157
Healthy Ageing Initiative	NA	Sweden	2012–18	ActiGraph GT3X (waist)	None	3793	70.4 (0.1)	1934 (51%)	4.3	138
Jackson Heart Study (JHS)	NA	USA	2000	Yamax SW-200 (waist)	None	401	60.2 (9.8)	244 (61%)	13.5	87
Nateglinide and Valsartan in Impaired Glucose Tolerance Outcomes Research (NAVIGATOR)	NA	40 countries	2002–04	Accusplit AE120 (waist)	None	7270	63.7 (6.9)	3698 (51%)	6.3	471

Data are n or n (%), unless otherwise stated. Mean data are presented with SD in parentheses. LFE=low-frequency extension. NA=not applicable.

* Unweighted mean age; weighted mean age was 56.9 years (SE 0.6).

## Research in context

### Evidence before this study

No evidence-based public health guidelines exist that recommend a specific number of steps per day for health benefits. We previously published a systematic review of the literature of daily steps and associations with all-cause mortality, cardiovascular disease, and dysglycaemia. Findings from prospective studies show mortality risk plateaus for older women (aged ≥62 years) at 7500 steps per day and among nationally representative samples of US and Norwegian adults at approximately 8000–12 000 steps per day. Observational studies have shown that stepping rate, a marker of intensity, is inversely associated with mortality; however, when adjusted for volume of steps per day, stepping rate is no longer significantly associated with mortality. A meta-analysis that used the effect estimates directly reported by seven publications found a linear association between step volume and mortality, observing large heterogeneity among studies and did not report associations by age, sex, or stepping rate. The Steps for Health Collaborative is an international consortium formed to determine the prospective association between device-measured step volume and rate with health outcomes, including mortality.

### Added value of this study

This meta-analysis of 15 prospective cohort studies from Asia, Australia, Europe, and North America (including 47 471 adults and 3013 deaths) provides evidence-based thresholds for the optimum number of steps per day associated with reduced risk of all-cause mortality. Each cohort study completed a standardised statistical analysis created by The Steps for Health Collaborative and these results were then meta-analysed. Compared with adults in the lowest steps per day quartile, adults in the highest steps per day quartile had a 40% to 53% lower risk of mortality. Taking more steps per day was associated with a progressively lower risk of all-cause mortality, up to a level that was similar by sex but varied by age. There was progressively lower risk of mortality among adults aged 60 years and older until about 6000–8000 steps per day and among adults younger than 60 years until about 8000–10 000 steps per day. We found inconsistent evidence that step intensity was associated with risk of mortality beyond total volume of steps.

### Implications of all the available evidence

Number of daily steps is a simple and feasible measure for monitoring and promoting physical activity globally as fitness trackers and mobile devices increase in popularity. Our findings suggest mortality benefits, particularly for older adults, can occur at levels less than the popular reference value of 10 000 steps per day. The findings from this meta-analysis can be used to inform step guidelines for public health promotion of physical activity.
