# Supplementary material for: Daily steps and all-cause mortality: a meta-analysis of 15 international cohorts
Source: Lancet Public Health. Author manuscript; Available in PMC 2022 Jul 18. (PMC9289978; doi:10.1016/S2468-2667(21)00302-9)
Supplement: MMC1 [file NIHMS1787838-supplement-MMC1.pdf]

THE LANCET
Public Health

Supplementary appendix

This appendix formed part of the original submission and has been peer reviewed. We post it as supplied by the authors.

Supplement to: Paluch AE, Bajpai S, Bassett DR, et al. Daily steps and all-cause mortality: a meta-analysis of 15 international cohorts. *Lancet Public Health* 2022; **7:** e219–28.

Supplementary Appendix

Daily Steps and Mortality: A Meta-Analysis of 15 International Cohorts

Table of Contents

Section 1. Study Level Characteristics Click to View
Table S1a. Study Level Descriptions of Data Collection and Processing……3
Table S1b. Study Level Detail Description of Covariates in Final Model……5
Table S1c. Study Level Descriptive Characteristics of Participants……6

Section 2. Risk of Bias Assessment Click to View
Table S2. Study Quality Assessment……10
New Castle Ottawa quality scale description……11

Section 3. Sensitivity Results for Fixed effects for Main Results Click to View
Table S3. Fixed vs Random Effect Models - Summary Results……12

Section 4. Overall Sample Supplement Results Click to View
Figure S1a. Overall Sample Quartile Comparisons, Forest Plots……13
Figure S1b. Overall Sample Quartile Comparisons, Funnel Plots……14
Figure S1c. Restricted Cubic Spline for Overall Sample……15

Section 5. Age-Stratified Supplement Results Click to View
Figure S2a. Age Subgroup Quartile Comparisons, Forest Plots……16
Figure S2b. Median Steps/d for Quartiles by Age Group Among Studies Included in Spline Analysis……18
Figure S2c. Spline Model using Same Referent Value for Both Younger and Older Adults……19

Section 6. Sex Stratified Supplement Results Click to View
Figure S3a. Sex Subgroup Quartile Comparisons, Forest Plots……20
Figure S3b. Median Steps/d for Quartiles by Sex Among Studies Included in Sex Stratified Spline Analysis……22
Figure S3c. Sex Stratified Restricted Cubic Spline……23

Section 7. Peak 30-Minute Stepping Rate supplement results Click to View
Figure S4a. Peak 30-Minute, Forest Plots……24
Figure S4b. Study Level Peak 30-Minute Medians by Quartile……24

Section 8. Peak 60-Minute Stepping Rate supplement results Click to View
Figure S5a. Peak 60-Minute, Forest Plots……25
Figure S5b. Study Level Peak 60-Minute Medians by Quartile……25

Section 9. Time spent at 40+ steps per minute supplement results Click to View
Figure S6a. Time Spent at ≥40 steps/min, Forest Plots……26
Figure S6b. Study Level Time Spent at ≥40 steps/min Medians by Quartile……26

Section 10. Time spent at 100+ steps per minute supplement results Click to View
Figure S7a. Time Spent at ≥100 steps/min, Forest Plots……27
Figure S7b. Study Level Time Spent at ≥100 steps/min Medians by……27

Section 11. Sensitivity results when removing deaths in first 2 years Click to View
Table S4. Sensitivity Results - Removing Deaths in First Two Years, Summary Results……28
Figure S8. Sensitivity Results - Removing Deaths in First Two Years, Forest Plots-……29

Section 12. Sensitivity results by years of follow-up (>6 or <6 years) Click to View
Figure S9. Stratified Results by Study with More or Less than Five Years of Follow-up, Forest Plots……30

Section 13. Sensitivity results by published vs non-published Click to View
Figure S10a. Stratified Results by Publication Status, Forest Plots……31
Figure S10b Stratified Results by Publication Status, Splines……32

Section 14. Sensitivity results for leave one out analysis Click to View
Table S5. Sensitivity Results - Leave-One-Study Out……33
Figure S11. Sensitivity Results - Leave-One-Device Out - Restricted Cubic Spline……33

Section 15. Study-level data request instructions and form……34

Table S1a. Characteristics of studies included in the meta-analysis –Descriptions of Data Collection and Processing

Study	Case Ascertainment	Device Type (wear location), Settings, Instructions	Stepping Metrics Included	Device Wear Criteria	Summary of Covariates in Final Model
Activity and Function in the Elderly in Ulm (ActiFE)	The local registration offices in Ulm, Germany	ActivPAL, PAL Technologies Ltd., Glasgow, UK (Thigh); 24 hours/day for 7 consecutive days; removed time from 12-6am	Peak 30 min.<br>Peak 60 min.<br>Time spent at 40+ steps per min.<br>Time spent at 100+ steps per min.	Device worn for 10h/d on ≥3 days	Age, sex, education, BMI, device wear time, smoking status, alcohol consumption, hypertension, diabetes, high cholesterol, CVD, cancer, five chair stand test
Atherosclerosis Risk in Communities (ARIC)	Community-wide hospital surveillance, records from national and local death registries, and death certificates.	ActiGraph wGT3X-BT (waist); 60 second epochs; 7 consecutive days during waking hours	Peak 30 min.<br>Time spent at 40+ steps per min.	Device worn for 10 hrs/day for ≥ 3 days	Age, sex, race/ethnicity, education, BMI, device wear time, smoking status, alcohol consumption, hypertension, diabetes, high cholesterol, CVD, cancer, self-rated health
Baltimore Longitudinal Study on Aging (BLSA)	Telephone follow-up, correspondence, and searches of the National Death Index	ActiGraph GT9X Link - LFE on (non-dominant wrist); 60 second epochs; 24-hour wear for 7 consecutive days; removed time from 12-6am;	Peak 30 min.<br>Peak 60 min.<br>Time spent at 40+ steps per min.<br>Time spent at 100+ steps per min.	Device worn for ≥ 3 days. Non-wear time < 10% of the remaining 18 wake hours, was defined as a valid day.	Age, sex, race/ethnicity, BMI, device wear time, CVD, hypertension, high cholesterol, cancer
British Regional Heart Study (BRHS)	The National Health Service central registers	ActiGraph GT3X (waist), 60 second epochs; 7 consecutive days during waking hours	None	Device worn for 10h/d on ≥4 days	Age, occupation, BMI, device wear time, smoking status, region of residence, alcohol consumption, duration of night sleep, CVD, mobility disability, living alone vs with others
Cancer Prevention Study-3 (CPS3)	U.S. National Death Index	Actigraph GT3X (waist); wake time only, collected at 1 sec epochs and aggregated to 60 sec epochs; 7 consecutive days during waking hours	None	Device worn for 10 hrs/day for ≥ 3 days	Age, sex, race/ethnicity, education, BMI, device wear time, smoking status, cancer, CVD, diabetes, respiratory disease
Coronary Artery Risk Development in Young Adults (CARDIA)	Medical records and death certificates	Actigraph 7164 (waist); 60 seconds epochs; 7 consecutive days during waking hours	Peak 30 min.<br>Peak 60 min.<br>Time spent at 40+ steps per min.<br>Time spent at 100+ steps per min.	Device worn for 10 hrs/day for ≥ 3 days	Age, sex, race/ethnicity, education, field center, BMI, device wear time, smoking status, healthy eating index, alcohol consumption, diabetes, hypertension, high cholesterol, CVD, self-rated health
Framingham Heart Study (FHS)	Death certificate. Additional information obtained from records supplied by hospital, physician, pathologist, medical examiner, or family	Actical model #198-0200-00; 30 seconds epochs (waist); During Generation 3- exam 2, participants instructed to wear the device 24 hours a day (removed time from 12-6am); for Gen 2-based exam 9 instructed to wear for wake time only	Peak 30 min.<br>Peak 60 min.<br>Time spent at 40+ steps per min.<br>Time spent at 100+ steps per min.	Device worn for 10 hrs/day for ≥ 3 days	Age, sex, cohort group, race/ethnicity, education, BMI, device wear time, smoking status, hypertension, high cholesterol, CVD, cancer , self-rated health
Healthy Ageing Initiative (HAI)	Swedish Cause of Death Register	ActiGraph GT3X (waist), 60 second epochs; 7 consecutive days during waking hours	None	Device worn for 10 hrs/day for ≥ 4 days	sex, education, income, BMI, device wear time, smoking status, marital status, hypertension, high cholesterol, CVD, cancer, antithrombotic agents, physical function

Additional studies on next page

BMI = Body Mass Index; CVD = Cardiovascular Disease; LFE = Low Frequency Extension

Table S1a cont. Characteristics of studies included in the meta-analysis –Descriptions of Data Collection and Processing

Study	Case Ascertainment	Device Type (wear location), Settings, Instructions	Stepping Metrics Included	Device Wear Criteria	Summary of Covariates in Final Model
Nateglinide and Valsartan in Impaired Glucose Tolerance Outcomes Research trial (NAVIGATOR)	Death certificates	Accusplit AE120 pedometer (waist) ; instructed to wear it during waking hours for 7 consecutive days. Participants were given a log book to write down their daily step count at the end of each day.	None	Steps per day recorded for 7 days	Age, sex, race/ethnicity, BMI, smoking status, CVD, diabetes, high cholesterol, cancer, emphysema,
National Health and Nutrition Examination Survey (NHANES)	U.S. National Death Index	Actigraph 7164 (waist); 60 seconds epochs, uniaxial; & 7 consecutive days during waking hours	Peak 30 min. Peak 60 min. Time spent at 40+ steps per min. Time spent at 100+ steps per min.	Device worn for 10 hrs/day for ≥ 3 days	Age, sex, race/ethnicity, education, BMI, device wear time, diet quality smoking status, alcohol consumption, self-rated health; mobility limitation; diabetes, CVD, cancer, chronic bronchitis, and emphysema
Niigata Elderly Study (NES)	Participants completed a health once a year. Contacted family member in case of missing health survey and confirmed date with death record.	Yamax EC-100S pedometer, YAMASA, Tokyo (waist); wear consecutively for 1 week during waking hours	None	Device worn for ≥ 3 days	Sex, education, BMI, smoking status, alcohol consumption, medication use
Norwegian National Physical Activity Surveillance 1 (NNPAS1)	The Norwegian Cause of Death Registry	ActiGraph GT1M (waist), non-LFE; collected at 10 sec, aggregated to 60 sec epochs; 7 consecutive days during waking hours	None	Device worn for 10 hrs/day for ≥ 4 days	Age, sex, education, BMI, device wear time, alcohol, smoking, diabetes, CVD, cancer, stroke; daily minutes of vigorous physical activity
Jackson Heart Study (JHS)	U.S. National Death Index	Yamax SW-200 pedometer (Yamax Corp., Tokyo, (waist). 3-day monitoring sessions that was repeated for a maximum of three separate occasions within a 6 month period (i.e. max 9 days total)	None	Device worn for ≥ 3 consecutive days for at least one of the assessment periods	Age, sex, education, BMI, hypertension, high cholesterol, smoking status, alcohol consumption, hypertension, diabetes, CVD
Tasped Pooled Cohort (Tasped)	The Australian National Death Index	Omron HJ-003 (waist), Omron HJ-102 (waist), and Yamax Digi-Walker (waist); 7 days at wake time	None	Device worn for ≥ 2 days, with at least one weekday	Age, sex, BMI, smoking status, alcohol consumption, stroke, myocardial infarction, hypertension, diabetes
Women's Health Study (WHS)	U.S. National Death Index, follow up with family members or postal authorities, with medical records, and death certificates.	Actigraph GT3X+ (waist); 30 Hz and aggregated into 60 seconds epochs; 7 consecutive days during wake time	Peak 30 min. Peak 60 min. Time spent at 40+ steps per min.	Device worn for 10h/d on ≥4 days	Age, BMI, device wear time, smoking status, alcohol consumption, hypertension, high cholesterol, CVD, diabetes, cancer, use of post-menopausal hormones, saturated fat intake

BMI = Body Mass Index; CVD = Cardiovascular Disease; LFE = Low Frequency Extension

Table S1b. Detailed Description of Covariates in Final Model

Study	Detailed Description of Covariates in Final Model
ActiFE	age, sex, education (years), device wear time, body mass index (kg/m2), smoking status (smoker, past smoker, non-smoker), alcohol consumption, stage 2 hypertension (Systolic pressure ≥ 140 mm Hg and/or diastolic pressure ≥ 90 mm Hg and/or told by a physician had hypertension), Diabetes (told by a physician or taking medication), high cholesterol (≥240 mg/dL), self-reported history of cardiovascular disease (myocardial infarction, heart failure, stroke), cancer (told by a physician), five chair raising test, estimated glomerular filtration rate (mL/min)
ARIC	age, sex, race/ethnicity, education (< high school, high school grad, > high school), body mass index (kg/m2), device wear time, smoking status (current smoker, non-smoker), alcohol consumption, Systolic blood pressure (mmHg), hypertension medications (yes/no), HDL cholesterol (mg/dL), LDL cholesterol (mg/dL), triglycerides (mg/dL), diabetes (fasting glucose ≥126 mg/dL or non-fasting ≥200 mg/dL, or taking diabetes medication or self reported diagnosis), cardiovascular disease (history of coronary heart disease, heart failure or stroke), self-rated health
BLSA	age, sex, race/ethnicity, education (years), body mass index (kg/m2), device wear time, cardiovascular disease (myocardial infarction, congestive heart failure, angina pectoris, bypass surgery or (balloon) angioplasty, or peripheral arterial disease), stage 2 hypertension (diagnosis of hypertension and taking antihypertensive medications), high cholesterol, and cancer (non-skin cancer).
BRHS	age, occupation (manual, non-manual labor), body mass index (kg/m2), device wear time, smoking (smoker, past, never), region of residence, alcohol consumption, hours of night-time sleep, cardiovascular disease (received a physician diagnosis of hear attack, heart failure or stroke), living along vs with others, mobility disability (Mobility disability was present if reported being unable to do any of: (1) walking 200 yards without stopping and without discomfort, (2) climbing a flight of 12 stairs without holding on and taking a rest, or (3) bending down and picking up a shoe from the floor)
CPS-3	age, sex, race/ethnicity, education (< high school, high school grad, ≥4 year college grad), body mass index (kg/m2), self-reported history of cancer, cardiovascular disease, diabetes or respiratory disease (yes/no)
CARDIA	age, sex, race/ethnicity, education (years), body mass index (kg/m2), device wear time, smoking status (current, former, never), alcohol consumption, healthy eating index score, stage 2 hypertension (systolic pressure ≥ 140 mm Hg and/or diastolic pressure is ≥ 90 mm Hg and/or taking medication), High cholesterol (≥200 mg/dL total cholesterol), Diabetes (fasting glucose ≥126 mg/dL or non-fasting ≥200 mg/dL, or taking diabetes medication), cardiovascular disease (history of coronary heart disease, heart failure or stroke), self-rated health
FHS	age, sex, race/ethnicity, education, body mass index (kg/m2), cohort, smoking status (current, non-smoker), alcohol consumption, stage 1 hypertension (systolic pressure ≥ 130 mm Hg and/or diastolic pressure is ≥ 80 mm Hg and/or taking medication), high cholesterol (self-reported taking medication to lower cholesterol), cardiovascular disease (myocardial infarction, stroke, heart failure), self-rated health
HAI	sex, education (primary, secondary, post-secondary), device wear time, body mass index (kg/m2), diabetes (self-reported or physician diagnosis), hypertension (≥ 140 mm Hg and/or diastolic pressure is ≥ 90 mm Hg or taking medication), high cholesterol (>240 mg/dL or taking medication), cancer diagnosis, martial status, household income, smoking status (current/non-smoker), physical function (timed-up-and-go test), cardiovascular disease diagnosis (myocardial infarction, heart failure, stroke)
NAVIGATOR	age, sex, race/ethnicity, body mass index (kg/m2), smoking status (current vs non smoker), alcohol consumption, cardiovascular disease (history of myocardial infarction, heart failure), emphysema, LDL cholesterol, cancer
NHANES	age, sex, race/ethnicity, body mass index (kg/m2), smoking status, alcohol consumption, mobility limitation (difficulty walking 0.25 miles, without special equipment, or up 10 steps), self-reported general health status, and self-reported diagnosis of the following conditions: diabetes, heart disease, stroke, heart failure, cancer, chronic bronchitis and emphysema
NES	sex, education (<11, 11, ≥12 years), body mass index (kg/m2), smoking status (never, past, current), alcohol consumption, medication use (yes/no taking any medications)
NNPAS	age, sex, device wear time, education, body mass index (kg/m2), smoking status (never, former, current), alcohol consumption, number of medical conditions, minutes/day of vigorous intensity physical activity
JHS	age, sex, education (< high school, high school graduate, > high school), body mass index (kg/m2), alcohol consumption, smoking status, stage 2 hypertension (Systolic pressure ≥ 140 mm Hg and/or diastolic pressure is ≥ 90 mm Hg and/or taking medication), high LDL cholesterol (≥ 160 mg/dL)
Tasped	age, sex, body mass index (kg/m2), education (< high school, high school grad, > 4 year college grad), study cohort, total energy intake (kJ/day), smoking status (current, non-smoker), alcohol consumption, stage 2 hypertension (systolic pressure ≥ 140 mm Hg and/or diastolic pressure is ≥ 90 mm Hg), high cholesterol (≥5.5 mmol/L), diabetes (reported), history of cardiovascular disease (reported history of stroke, myocardial infarction)
WHS	age, device wear time, body mass index (kg/m2), smoking status (current/non-smoker), alcohol consumption, dietary intake (intake of saturated fat, fiber, fruits and vegetables), hormone therapy, parental history of myocardial infarction, family history of cancer, self-rated health, cardiovascular disease diagnosis, cancer diagnosis, cancer screening, self-reported hypertension, self-reported high cholesterol, self-reported diabetes

Table S1c. Descriptive characteristics of participants

	ActiFE		ARIC		BLSA		BRHS
	Men	Women	Women	Men	Men	Women	Men
N	529	712	266	186	181	201	1397
Age (y), mean (SD)	75.9 (6.4)	74.8 (6.5)	78.0 (4.5)	79.1 (4.8)	77 (9.0)	75.4 (8.8)	78.4 (4.6)
BMI (kg/m²), mean (SD)	27.8 (3.6)	27.3 (4.6)	27.7 (5.3)	28.5 (4.4)	27.4 (3.9)	26.4 (4.9)	27.1 (3.8)
BMI Categories, n (%)							
BMI < 24.9 kg/m²	145 (21)	159 (30)	80 (31)	42 (23)	51 (28)	84 (46)	402 (29)
BMI: 25.0-29.9 kg/m²	394 (55)	230 (44)	102 (40)	81(44)	89 (28.20)	74 (37)	722 (52)
BMI ≥30.0 kg/m²	170 (24)	132 (25)	75 (29)	61 (33)	40 (22.1)	40 (20)	270 (19)
race/ethnicity, n (%)							
Non-Hispanic White	>99%	>99%	202 (76)	159 (85)	139 (77)	132 (66)	>99%
Non-Hispanic Black	-	-	64 (24)	27 (15)	34 (19)	57 (28)	-
Asian	-	-	-	-	8 (4)	11 (6)	-
Hispanic	-	-	-	-	-	-	-
Others	-	-	-	-	-	1 (0.5)	-
Hypertension (Stage 2), n (%)	582 (82)	401 (77)	197 (74)	143 (77)	89 (49)	101 (50)	1212 (87)
High total cholesterol, n (%)	90 (13)	179 (33.8)	104 (39)	29 (16)	106 (59)	120 (60)	796 (58)
Diabetes, n (%)	113 (21)	58 (8)	62 (23)	61 (33)	32 (18)	37 (18)	215 (16)
History of Cardiovascular disease, n (%)	197 (28)	105 (20)	23 (9)	55 (30)	28 (16)	9 (5)	217 (16)
History of Cancer, n (%)	148 (20.8)	79 (15)	0	1 (0.5)	81 (45)	57 (28)	-
Average Steps/day, mean (SD)	7815 (3529)	7761 (3282)	3353 (1760)	3580 (1841)	19660 (4132)	20784 (4176)	4869 (2764)
No. of days of compliant device wear, mean (SD)	-	-	7.1 (2.4)	6.7 (1.9)	5.9 (0.4)	6.0 (0.3)	6.7 (0.8)
Minutes/day of device wear, mean (SD)	-	-	847 (102)	849 (89)	1064 (13)	1061 (11)	853 (68)

BMI = Body Mass Index, measured by height and weight during study examinations

Table S1c continued. Descriptive characteristics of participants

	CPS-3		CARDIA		FHS		HAI	
	**Men**	**Women**	**Men**	**Women**	**Men**	**Women**	**Men**	**Women**
N	292	428	907	1203	2104	2444	1859	1934
Age (y), mean (SD)	53.5 (10.3)	52.1 (9.7)	45.2 (3.6)	45.2 (3.6)	55.5 (14.0)	55.2 (13.8)	70.4 (0.1)	70.4 (0.1)
BMI (kg/m²), mean (SD)	27 (4.7)	26.9 (5.8)	29.0 (7.0)	29.2 (7.4)	28.8 (4.7)	27.4 (5.7)	26.7 (3.7)	26.3 (4.6)
BMI Categories, n (%)								
BMI < 24.9 kg/m²	100 (34)	197 (46)	632 (30)	413 (34)	424 (20)	977 (40)	616 (33)	863 (45)
BMI: 25.0-29.9 kg/m²	130 (45)	127 (30)	739 (35)	332 (28)	976 (46)	814 (33)	926 (50)	717 (37)
BMI ≥30.0 kg/m²	62 (21)	104 (24)	739 (35)	460 (38)	704 (36)	653 (27)	317 (17)	354 (18)
race/ethnicity, n (%)								
Non-Hispanic White	197 (67)	280 (65)	1222 (57.91)	649 (53.86)	1902 (90)	2183 (89)	>99%	>99%
Non-Hispanic Black	52 (18)	83 (19)	888 (42.09)	556 (46.12)	-	-	-	-
Asian	-	-	-	-	-	-	-	-
Hispanic	43 (15)	65 (15)	-	-	-	-	-	-
Others	-	-	-	-	-	-	-	-
Hypertension (Stage 2), n (%)	104 (46)	118 (53)	413 (20)	248 (25)	1308 (62)	1113 (46)	1533 (83)	1537 (80)
High total cholesterol, n (%)	136 (52)	123 (48)	1189 (56)	568 (47)	758 (36)	607 (25)	1152 (62)	1275 (66)
Diabetes, n (%)	26 (9)	19 (4)	166 (8)	96 (8)	-	-	215 (12)	127 (7)
History of Cardiovascular disease, n (%)	19 (7)	7 (2)	25 (1)	11 (1)	203 (10)	122 (5)	414 (22)	172 (9)
History of Cancer, n (%)	15 (5)	7 (2)	-	-	281 (13)	363 (15)	355 (19)	348 (18)
Average Steps/day, mean (SD)	7840 (3124)	7544 (2806)	9812 (3245)	9174 (2920)	7721 (4072)	6869 (3551)	7116 (3060)	7153 (3169)
No. of days of compliant device wear, mean (SD)	12.4 (2.7)	12.01 (3.12)	7.0 (1.0)	6.9 (1.3)	7.4 (1.32)	7.2 (1.4)	7.0 (0.9)	7.0 (0.8)
Minutes/day of device wear, mean (SD)	919 (68)	910 (64)	872 (86)	855 (83)	915 (94)	905 (91)	886 (103)	872 (93)

BMI = Body Mass Index, measured by height and weight during study examinations

Table S1c continued. Descriptive characteristics of participants

	JHS		NHANES		NAVIGATOR		NES	
	Women	Men	Women	Men	Women	Men	Men	Women
N	244	157	1189	1193	3698	3573	227	189
Age (y), mean (SD)	61.0 (9.3)	59.0 (10.5)	57.3	56.5	63.6 (7.7)	63.9 (7.0)	71.0 (0)	71.0 (0)
BMI (kg/m^2), mean (SD)	31.9 (7.1)	29.4 (6.2)	29.2	29	31.2 (5.9)	29.6 (4.6)	22.4 (2.8)	22.9 (3.1)
BMI Categories, n (%)								
BMI < 24.9 kg/m^2	28 (11)	28 (18)	358 (35)	294 (22)	466 (13)	452 (13)	166 (73)	132 (70)
BMI: 25.0-29.9 kg/m^2	78 (32)	67 (43)	333 (27)	495 (42)	1265 (34)	1669 (47)	39 (17)	40 (21)
BMI ≥30.0 kg/m^2	138 (57)	62 (39)	490 (38)	395 (36)	47 (53)	1450 (41)	2 (0.9)	4 (2.1)
race/ethnicity, n (%)								
Non-Hispanic White	-	-	643 (77)	667 (78)	2956 (80)	3006 (84)	-	-
Non-Hispanic Black	244 (100)	157 (100)	282 (11)	263 (10)	101 (3)	65 (2)	-	-
Asian	-	-	-	-	270 (7)	276 (6)	227 (100)	189 (100)
Hispanic	-	-	220 (8)	228 (8)			-	-
Others	-	-	44 (4)	35 (5)	371 (10)	226 (4)	-	-
Hypertension (Stage 2), n (%)	166(68)	90 (57)	572 (44)	514(38.7)	2998 (81)	2677 (75)	-	-
High total cholesterol, n (%)	40 (16)	22 (14)	469 (39)	482 (42)	1604 (43)	1656 (46)	-	-
Diabetes, n (%)	-	-	208 (13)	200 (13)	1530 (41)	1191 (33)	-	-
History of Cardiovascular disease, n (%)	-	-	45 (3)	101 (8)	119 (3)	122 (3)	-	-
History of Cancer, n (%)	-	-	165 (14)	134 (10)	74 (2)	57 (2)	-	-
Average Steps/day, mean (SD)	4941 (3693)	6386 (4031)	7569 (3690)	8565 (4175)	6600 (4803)	5945 (3961)	6589 (2913)	6332 (2539)
No. of days of compliant device wear, mean (SD)	-	-	6.6	6.6	6.6 (1)	6.3 (1)	-	-
Minutes/day of device wear, mean (SD)	-	-	-	-	-	-	-	-

BMI = Body Mass Index, measured by height and weight during study examinations

Table S1c continued. Descriptive characteristics of participants

	NNPAS1		Tasped		WHS
	Men	Women	Men	Women	Women
N	1416	1627	1226	1350	16741
Age (y), mean (SD)	50.6 (14.8)	49.2 (14.9)	59.8 (13.0)	57.8 (13.4)	72.0 (5.7)
BMI (kg/m^2), mean (SD)	26.2 (3.5)	24.8 (4.2)	27.4 (4.1)	27.3 (4.1)	26.2 (5.0)
BMI Categories, n (%)					
BMI < 24.9 kg/m^2	577 (41)	976 (60)	328 (27)	496 (37)	7746 (46)
BMI: 25.0-29.9 kg/m^2	649 (46)	477 (29)	623 (51)	509 (38)	5731 (34.23)
BMI ≥30.0 kg/m^2	190 (13)	174 (11)	273 (22)	339 (25)	3261 (19.48)
race/ethnicity, n (%)					
Non-Hispanic White	>99%	>99%	>99%	>99%	15952 (95)
Non-Hispanic Black	-	-	-	-	252 (1.5)
Asian	-	-	-	-	197 (1.2)
Hispanic	-	-	-	-	151 (0.9)
Others	-	-	-	-	54 (0.3)
Hypertension (Stage 2), n (%)	-	-	413 (34)	517 (38)	11436 (68)
High total cholesterol, n (%)	-	-	728 (59)	694 (51)	12280 (73)
Diabetes, n (%)	74 (5)	37 (2)	83 (7)	79 (6)	1502 (9)
History of Cardiovascular disease, n (%)	152 (11)	106 (7)	-	-	400 (2)
History of Cancer, n (%)	27 (2)	27 (2)	-	-	1991 (12)
Average Steps/day, mean (SD)	7934 (2960)	8135 (3002)	9554 (5014)	9720 (4951)	5499 (2663)
No. of days of compliant device wear, mean (SD)	6.8 (1.0)	6.8 (0.9)	-	-	6.7 (0.6)
Minutes/day of device wear, mean (SD)	878 (64)	872 (61)	-	-	892 (75)

BMI = Body Mass Index, measured by height and weight during study examinations

Table S2: Study Quality Assessment

Newcastle-Ottawa Quality Assessment of Studies									
Study		Selection			Comparability	Outcome			Overall Score
	Representativeness	Selection	Ascertainment Exposure	Outcome		Assessment	Follow-Up	Adequacy	
ActiFE-Ulm	B*	A*	A*	A*	A* B*	B *	A*	A*	9
ARIC	B*	A*	A*	A*	A* B*	B *	A*	A*	9
BLSA	B*	A*	A*	A*	A* B*	B *	A*	A*	9
BRHS	C	A*	A*	A*	A* B*	B *	A*	A*	8
CARDIA	B*	A*	A*	A*	A* B*	B *	A*	A*	9
CPS-3	B*	A*	A*	A*	A* B*	B *	A*	A*	9
FHS	B*	A*	A*	A*	A* B*	B *	A*	A*	9
NES	B*	A*	C	A*	A* B*	B *	A*	A*	8
JHS	B*	A*	C	A*	A* B*	B *	A*	A*	8
HAI	B*	A*	A*	A*	A* B*	B *	A*	A*	9
NAVIGATOR	C	A*	C	A*	A* B*	B *	A*	A*	7
NHANES	A*	A*	A*	A*	A* B*	B *	A*	A*	9
Norwegian Surveillance	A*	A*	A*	A*	A* B*	B *	A*	A*	9
Tasped	B*	A*	C	A*	A* B*	B *	A*	A*	8
WHS	C	A*	A*	A*	A* B*	B *	A*	A*	8

A study can be awarded a maximum of one star for each numbered item within the Selection and Outcome categories. A maximum of two stars can be given for Comparability. Overall score sums the number of stars.

Newcastle-Ottawa Quality Assessment Scale Description

COHORT STUDIES

Note: A study can be awarded a maximum of one star for each numbered item within the Selection and Outcome categories. A maximum of two stars can be given for Comparability

Selection

1) Representativeness of the exposed cohort
   a) truly representative of the average adult in the community ‾
   b) somewhat representative of the average adult in the community ‾
   c) selected group of users e.g. nurses, volunteers, only those with a precondition/morbidity, only men or only women
   d) no description of the derivation of the cohort
2) Selection of the non exposed cohort
   a) drawn from the same community as the exposed cohort ‾
   b) drawn from a different source
   c) no description of the derivation of the non exposed cohort
3) Ascertainment of exposure
   a) secure record (eg surgical records) ‾
   b) structured interview ‾
   c) written self report (if participants reported their steps per day on a log)
   d) no description
4) Demonstration that outcome of interest was not present at start of study
   a) yes ‾
   b) no

Comparability

1) Comparability of cohorts on the basis of the design or analysis
   a) study controls for AGE (select the most important factor) ‾
   b) study controls for any additional factor (gender, SES, and BMI) ‾

Outcome

1) Assessment of outcome
   a) independent blind assessment ‾
   b) record linkage ‾
   c) self report
   d) no description
2) Was follow-up long enough for outcomes to occur
   a) yes (select an adequate follow up period for outcome of interest) ‾
   b) no
3) Adequacy of follow up of cohorts
   a) complete follow up - all subjects accounted for ‾
   b) subjects lost to follow up unlikely to introduce bias - small number lost - > ____ % (select an adequate %) follow up, or description provided of those lost) ‾
   c) follow up rate < ____% (select an adequate %) and no description of those lost
   d) no statement

Table S3. Fixed vs Random Effects Models

Primary Results Fixed vs. Random Effects

	HR	95% CI
Quartile 2 vs Quartile 1 (ref)		
Fixed	0.62	(0.56; 0.69)
Random	0.60	(0.51; 0.71)
Quartile 3 vs Quartile 1 (ref)		
Fixed	0.55	(0.49; 0.61)
Random	0.55	(0.49; 0.62)
Quartile 4 vs Quartile 1 (ref)		
Fixed	0.49	(0.43; 0.56)
Random	0.47	(0.38; 0.57)

k=14 studies (all included)

Hazard Ratio and 95% Confidence Intervals [HR (95% CI)] adjusted for age, accelerometer wear time, race/ethnicity (if applicable), sex (if applicable), education or occupation, BMI, and study-specific variables for lifestyle, chronic conditions or risk factors, and general health status (see supplement table 1a).

Figure S1a: Overall Sample Quartile Comparisons – Forest Plots

Study	TE	seTE	HR(95%CI)	n	deaths	weight
BRHS	-0.39	0.1768	0.68 [0.48; 0.96]	349	60	9.4%
CPS-3	0.14	1.5902	1.15 [0.05; 25.96]	180	2	0.3%
FHS	-0.61	0.2391	0.55 [0.34; 0.87]	1137	25	7.0%
NNPAS1	-0.73	0.2926	0.48 [0.27; 0.85]	761	17	5.5%
ActiFE	-0.31	0.1447	0.73 [0.55; 0.97]	310	84	10.8%
WHS	-0.58	0.1236	0.56 [0.44; 0.71]	4185	103	11.8%
CARDIA	-1.41	0.4311	0.24 [0.11; 0.57]	528	7	3.1%
NHANES	-0.97	0.1579	0.38 [0.28; 0.52]	596	112	10.2%
BLSA	-0.97	0.6787	0.38 [0.10; 1.44]	95	5	1.4%
NES	-0.04	0.3228	0.96 [0.51; 1.82]	104	20	4.8%
JHS	-0.23	0.2819	0.79 [0.46; 1.38]	100	25	5.8%
Tasped	-0.48	0.1884	0.62 [0.43; 0.90]	644	44	8.9%
NAVIGATOR	-0.17	0.1170	0.84 [0.67; 1.06]	1827	133	12.1%
ARIC	-1.27	0.6980	0.28 [0.07; 1.10]	113	3	1.4%
HAI	-0.43	0.2213	0.65 [0.42; 1.00]	948	36	7.6%
**Random effects model**			**0.60 [0.51; 0.71]**			**100.0%**

Heterogeneity: $I^2 = 52\%$, $\tau^2 = 0.0462$, $p = 0.01$

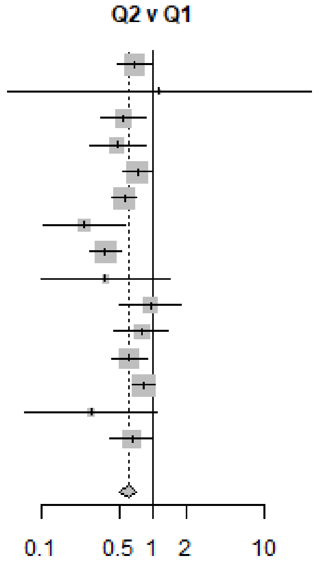

Study	TE	seTE	HR(95%CI)	n	deaths	weight
BRHS	-0.65	0.2011	0.52 [0.35; 0.77]	350	42	8.1%
CPS-3	-0.43	1.7569	0.65 [0.02; 20.34]	180	1	0.1%
FHS	-0.74	0.2732	0.48 [0.28; 0.82]	1137	19	4.7%
NNPAS1	-0.78	0.2992	0.46 [0.26; 0.83]	761	16	4.0%
ActiFE	-0.43	0.1657	0.65 [0.47; 0.90]	310	84	11.2%
WHS	-0.74	0.1451	0.48 [0.36; 0.63]	4186	77	13.8%
CARDIA	-0.53	0.3230	0.59 [0.31; 1.10]	528	17	3.4%
NHANES	-0.73	0.1207	0.48 [0.38; 0.61]	596	58	18.0%
BLSA	-1.39	0.8312	0.25 [0.05; 1.27]	96	2	0.5%
NES	0.29	0.3034	1.34 [0.74; 2.43]	104	25	3.9%
JHS	-0.71	0.3235	0.49 [0.26; 0.93]	100	16	3.4%
Tasped	-0.43	0.2101	0.65 [0.43; 0.98]	644	37	7.5%
NAVIGATOR	-0.49	0.1412	0.61 [0.46; 0.80]	1822	82	14.4%
ARIC	-1.27	0.6519	0.28 [0.08; 1.00]	113	3	0.9%
HAI	-0.60	0.2352	0.55 [0.35; 0.87]	950	32	6.1%
**Random effects model**			**0.55 [0.49; 0.62]**			**100.0%**

Heterogeneity: $I^2 = 12\%$, $\tau^2 = 0.0065$, $p = 0.32$

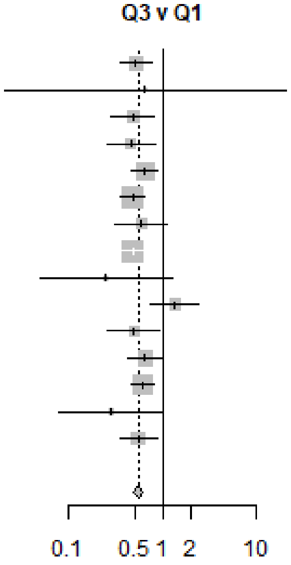

Study	TE	seTE	HR(95%CI)	n	deaths	weight
BRHS	-1.02	0.2559	0.36 [0.22; 0.59]	352	24	8.0%
CPS-3	-0.04	1.8020	0.96 [0.03; 32.82]	180	1	0.3%
FHS	-0.41	0.2694	0.66 [0.39; 1.12]	1137	23	7.6%
NNPAS1	-0.89	0.3412	0.41 [0.21; 0.80]	761	14	5.7%
ActiFE	-0.46	0.1826	0.63 [0.44; 0.90]	310	48	10.8%
WHS	-1.12	0.1812	0.33 [0.23; 0.47]	4185	49	10.8%
CARDIA	-0.72	0.3341	0.49 [0.25; 0.94]	527	16	5.9%
NHANES	-0.82	0.1768	0.44 [0.31; 0.62]	595	53	11.0%
BLSA	-0.51	0.7813	0.60 [0.13; 2.77]	95	3	1.5%
NES	-0.79	0.3891	0.45 [0.21; 0.97]	104	10	4.7%
JHS	-0.99	0.3907	0.37 [0.17; 0.80]	101	10	4.7%
Tasped	-0.37	0.2338	0.69 [0.44; 1.09]	644	31	8.8%
NAVIGATOR	-0.37	0.1455	0.69 [0.52; 0.92]	1807	82	12.4%
ARIC	-0.94	0.6117	0.39 [0.12; 1.29]	113	5	2.3%
HAI	-1.66	0.3472	0.19 [0.10; 0.38]	948	10	5.6%
**Random effects model**			**0.47 [0.39; 0.57]**			**100.0%**

Heterogeneity: $I^2 = 47\%$, $\tau^2 = 0.0599$, $p = 0.02$

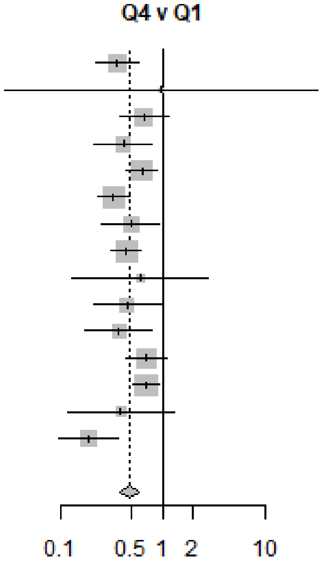

TE = treatment effect (log hazard ratio); seTE = standard error of treatment estimate; Q = Quartile; HR (95% CI) = Hazard Ratio and 95% Confidence Intervals.
Models adjusted for age, accelerometer wear time, race/ethnicity (if applicable), sex (if applicable), education or occupation, BMI, and study-specific variables for lifestyle, chronic conditions or risk factors, and general health status (see supplement 1a).

Figure S1b: Overall Sample Quartile Comparisons – Funnel Plots

Quartile 2 v 1

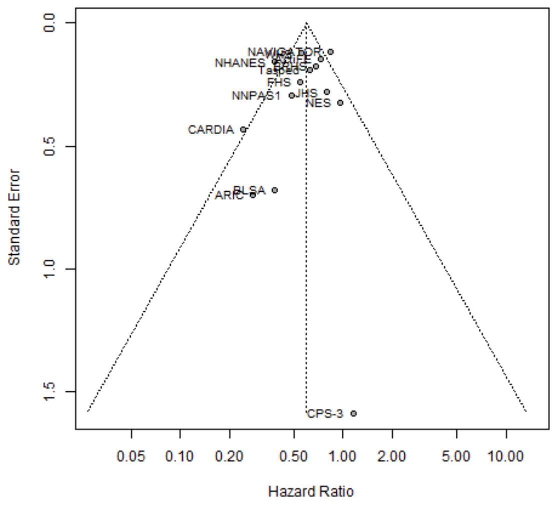

Quartile 3 v 1

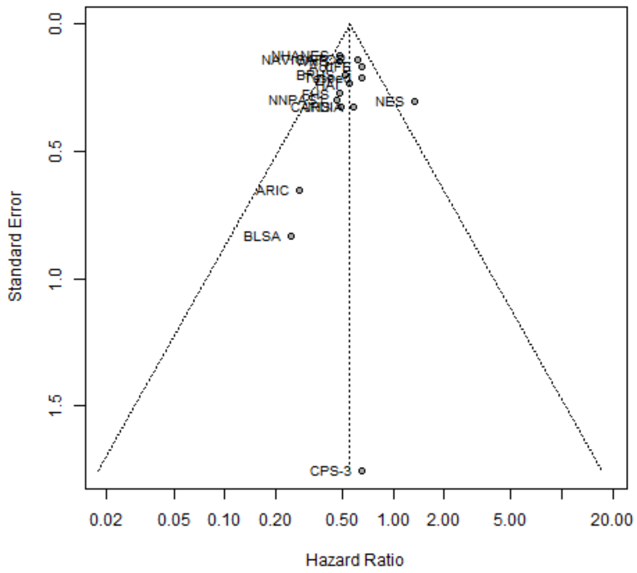

Quartile 4 v 1

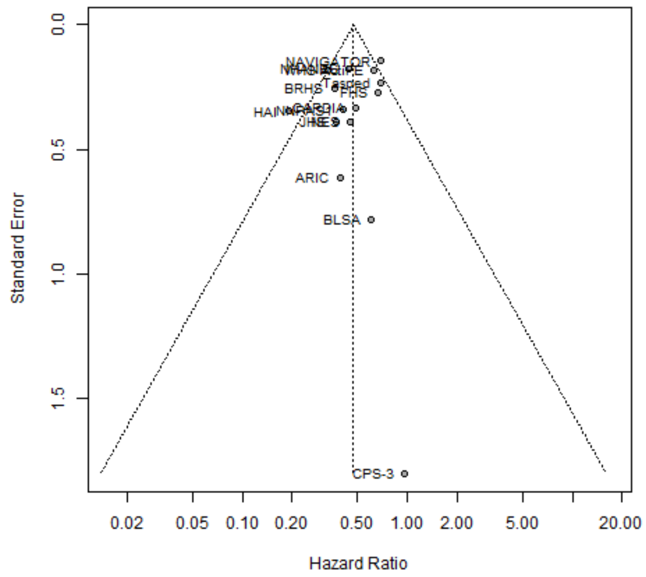

Figure S1c
Restricted Cubic Spline for Overall Sample – Final adjusted Model

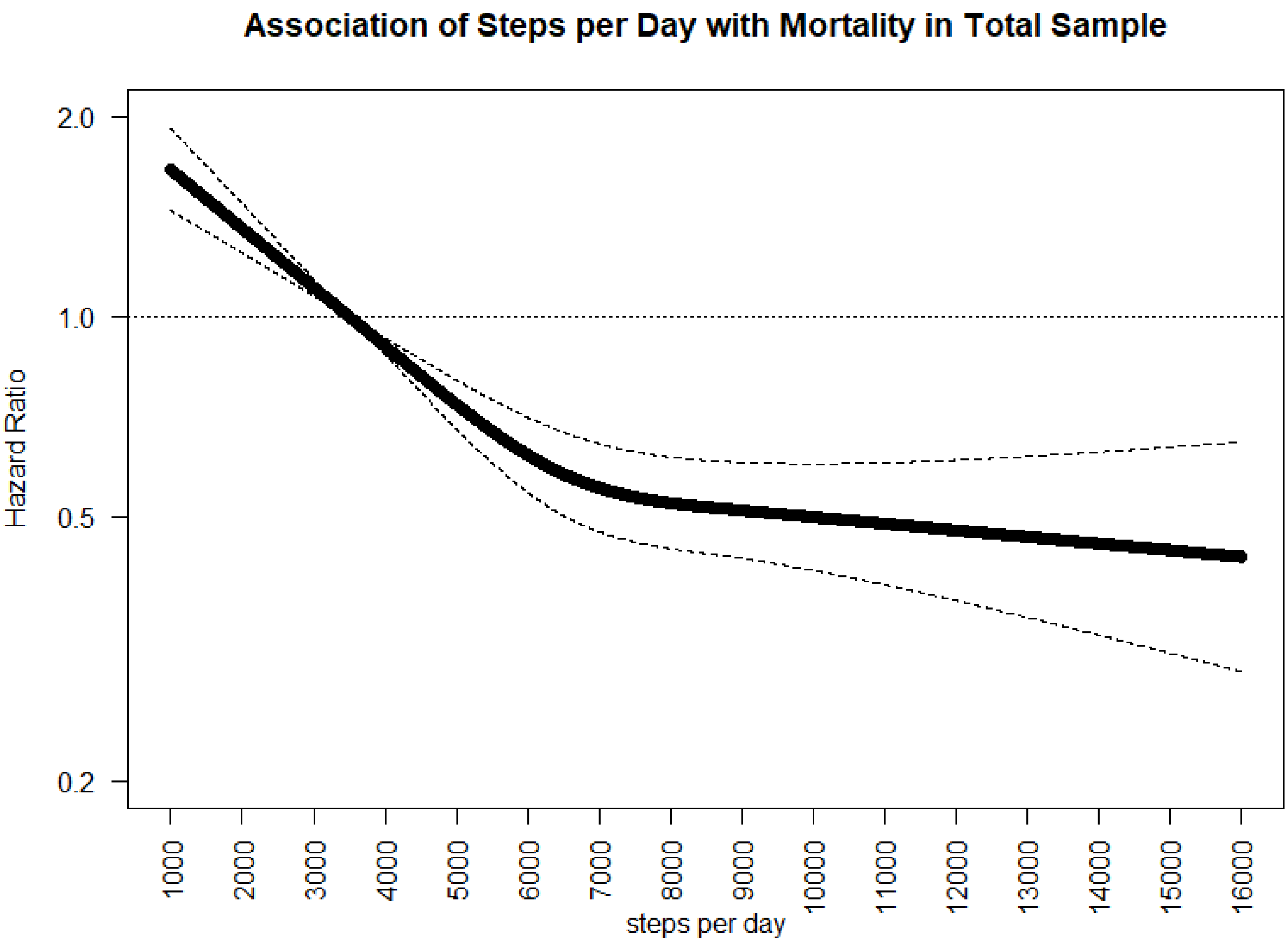

Hazard Ratio and 95% Confidence Intervals [HR (95% CI)] adjusted for age, accelerometer wear time, race/ethnicity (if applicable), sex (if applicable), education or occupation, BMI, and study-specific variables for lifestyle, chronic conditions or risk factors, and general health status (see supplement). Reference set 3,500 steps/d (median of the lowest quartile median).
K=14 studies (excludes BLSA)

Figure S2a: Association of Steps/Day with All-Cause Mortality Stratified by Younger (<60 years) and Older (≥ 60 years) Adults – Forest Plots

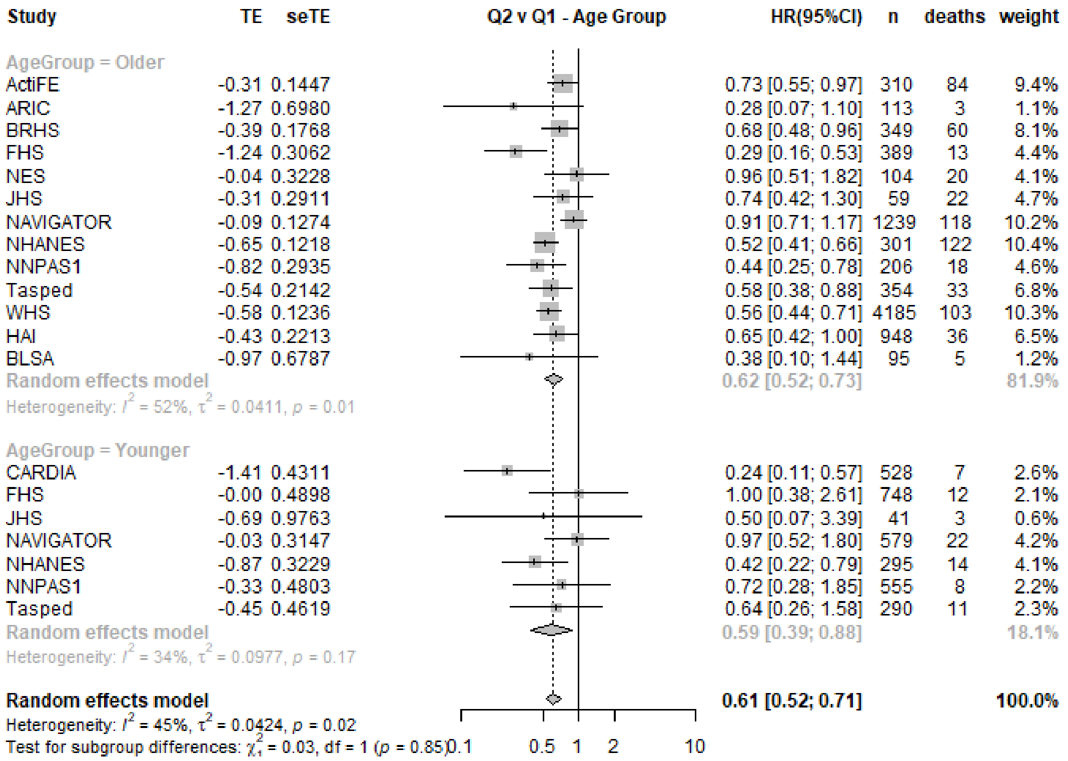

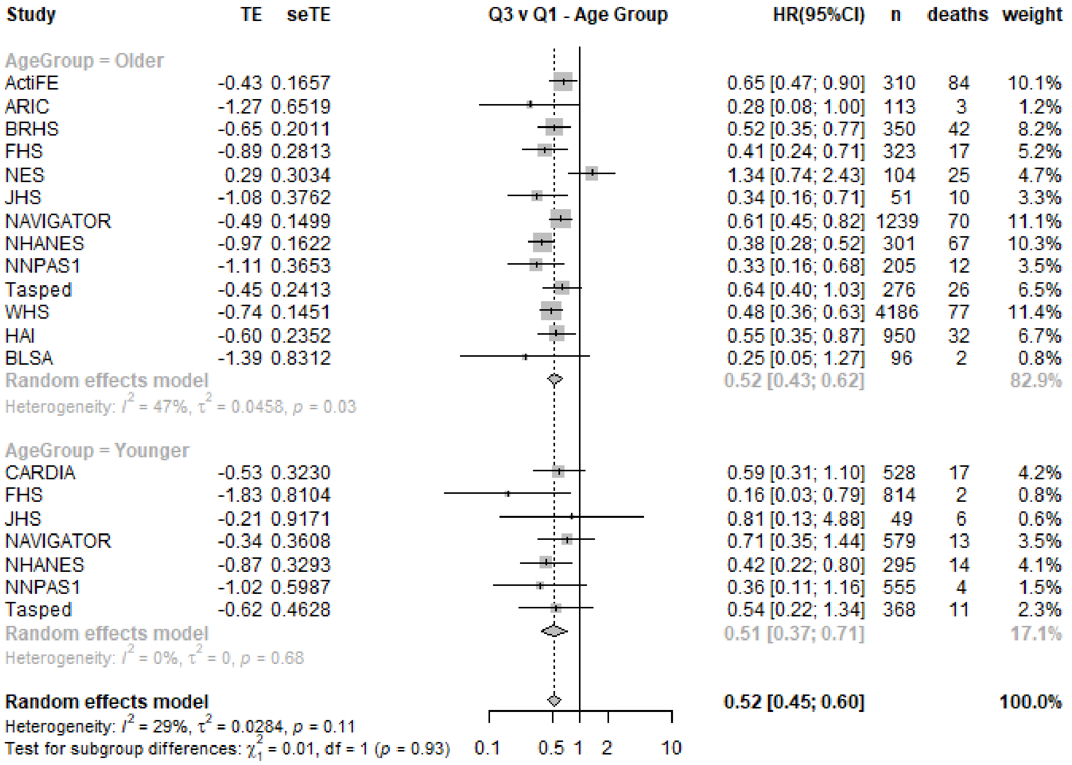

Figure S2a cont: Association of Steps/Day with All-Cause Mortality Stratified by Younger (<60 years) and Older (≥ 60 years) Adults – Forest Plots

Study	TE	seTE	HR(95%CI)	n	deaths	weight
AgeGroup = Older						
ActiFE	-0.46	0.1826	0.63 [0.44; 0.90]	310	48	9.4%
ARIC	-0.94	0.6117	0.39 [0.12; 1.29]	113	5	2.0%
BRHS	-1.02	0.2559	0.36 [0.22; 0.59]	352	24	6.9%
FHS	-0.78	0.3111	0.46 [0.25; 0.84]	251	14	5.5%
NES	-0.79	0.3891	0.45 [0.21; 0.97]	104	10	4.1%
JHS	-1.11	0.3974	0.33 [0.15; 0.72]	39	9	4.0%
NAVIGATOR	-0.49	0.1592	0.61 [0.45; 0.83]	1239	65	10.3%
NHANES	-1.02	0.1868	0.36 [0.25; 0.52]	300	49	9.3%
NNPAS1	-1.20	0.4295	0.30 [0.13; 0.70]	206	9	3.5%
Tasped	-0.30	0.2882	0.74 [0.42; 1.30]	193	18	6.1%
WHS	-1.12	0.1812	0.33 [0.23; 0.47]	4185	49	9.5%
HAI	-1.66	0.3472	0.19 [0.10; 0.38]	948	10	4.8%
BLSA	-0.51	0.7813	0.60 [0.13; 2.77]	95	3	1.3%
Random effects model			0.43 [0.34; 0.53]			76.7%
Heterogeneity: $I^2 = 48\%$, $\tau^2 = 0.0647$, $p = 0.03$						
AgeGroup = Younger						
CARDIA	-0.72	0.3341	0.49 [0.25; 0.94]	527	16	5.1%
FHS	-0.44	0.5340	0.64 [0.23; 1.83]	886	9	2.5%
JHS	-2.06	1.2985	0.13 [0.01; 1.63]	62	1	0.5%
NAVIGATOR	0.08	0.3454	1.08 [0.55; 2.13]	579	18	4.8%
NHANES	-0.71	0.3459	0.49 [0.25; 0.97]	295	24	4.8%
NNPAS1	-0.56	0.5321	0.57 [0.20; 1.62]	556	7	2.5%
Tasped	-0.60	0.4628	0.55 [0.22; 1.36]	451	13	3.1%
Random effects model			0.60 [0.44; 0.83]			23.3%
Heterogeneity: $I^2 = 0\%$, $\tau^2 = 0$, $p = 0.53$						
**Random effects model**			**0.46 [0.39; 0.55]**			**100.0%**
Heterogeneity: $I^2 = 39\%$, $\tau^2 = 0.0571$, $p = 0.04$						
Test for subgroup differences: $\chi^2_1 = 3.11$, df = 1 ($p = 0.08$)						

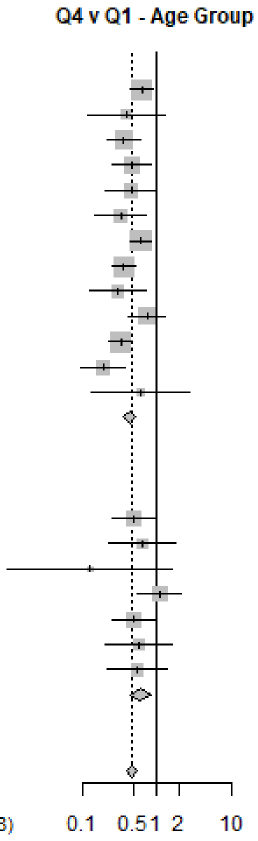

TE = treatment effect (log hazard ratio); seTE = standard error of treatment estimate; Q = Quartile; HR (95% CI) = Hazard Ratio and 95% Confidence Intervals.
Models adjusted for age, accelerometer wear time, race/ethnicity (if applicable), sex (if applicable), education or occupation, BMI, and study-specific variables for lifestyle, chronic conditions or risk factors, and general health status (see supplement 1a).

Figure S2b. Median Steps/d for Quartiles by Age Group Among Studies Included in Spline Analysis

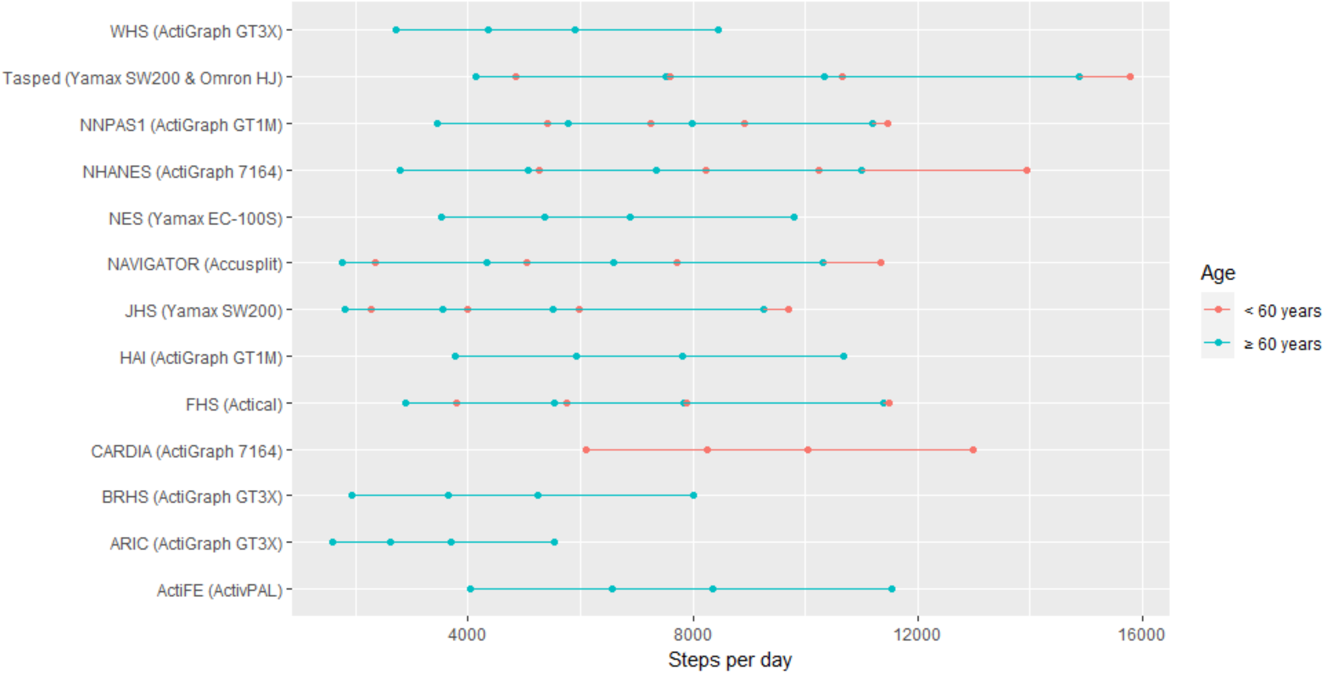

Figure S2c
Spline Model using Same Referent Value for Both Younger and Older Adults

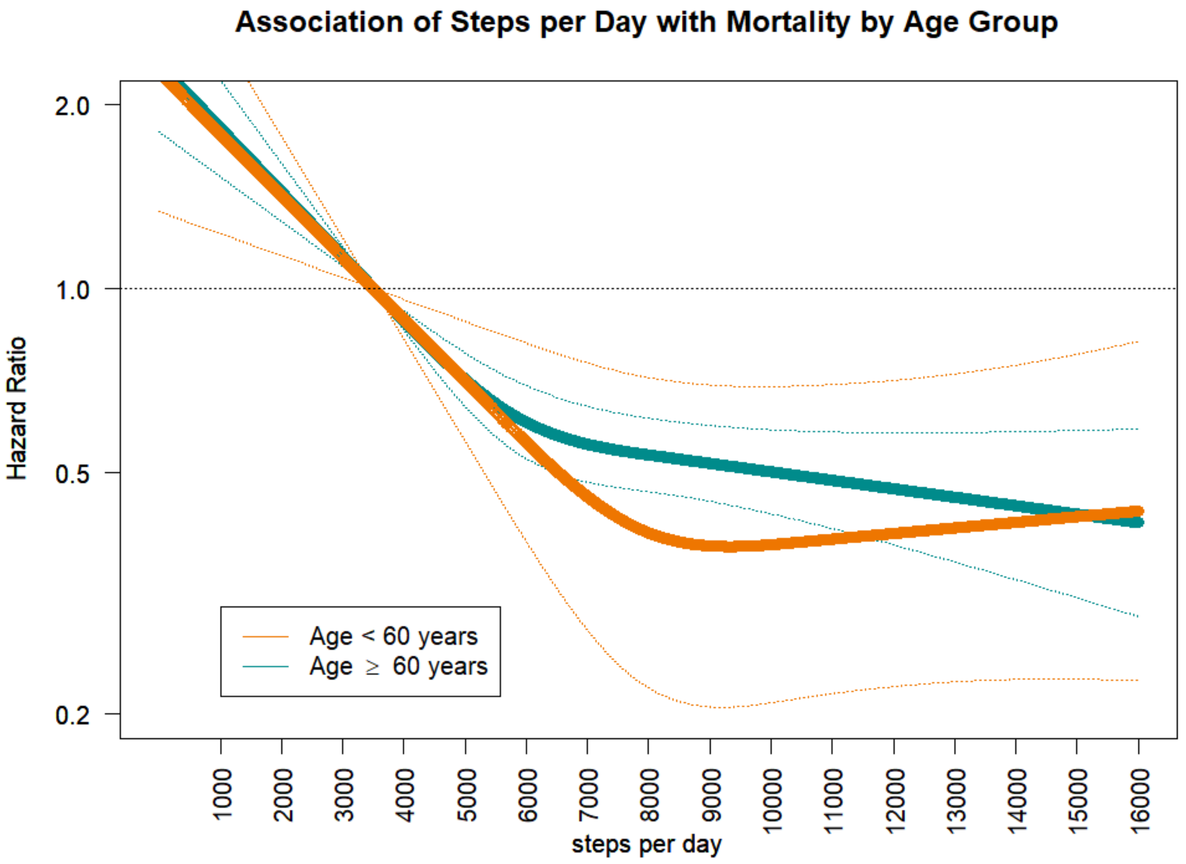

Restricted Cubic Splines using the same referent value for both age groups (median of the lowest quartile of 3,500 steps for overall sample) demonstrated the same plateauing at 6,000-8,000 for older adults and 8,000-10,000 steps/d for younger adults, as seen in primary result figure.

Three knots set at 25, 50, and 75 percentiles. Models adjusted for age, accelerometer wear time, race/ethnicity (if applicable), sex (if applicable), education or occupation, BMI, and study-specific variables for lifestyle, chronic conditions or risk factors, and general health status (see supplement 1a).

Figure S3a. Association of Steps/Day with All-Cause Mortality Stratified by Sex – Forest Plots

Q2 v Q1 - By Sex

Study	TE	seTE	HR(95%CI)	n	deaths	weight
**Sex = Men**						
ActiFE	-0.20	0.1813	0.82 [0.57; 1.17]	177	60	7.0%
BRHS	-0.39	0.1768	0.68 [0.48; 0.96]	349	60	7.2%
CARDIA	-0.90	0.5423	0.41 [0.14; 1.17]	214	5	1.7%
FHS	-0.80	0.3132	0.45 [0.24; 0.83]	506	14	4.0%
NES	0.22	0.3568	1.25 [0.62; 2.51]	56	18	3.3%
JHS	-0.56	0.4110	0.57 [0.26; 1.28]	40	12	2.7%
NNPAS1	-1.11	0.3893	0.33 [0.15; 0.71]	354	9	3.0%
Tasped	-0.54	0.2394	0.58 [0.36; 0.93]	303	27	5.5%
NHANES	-0.73	0.1207	0.48 [0.38; 0.61]	298	71	8.9%
NAVIGATOR	-0.27	0.1505	0.76 [0.57; 1.02]	893	79	8.0%
HAI	-0.33	0.2741	0.72 [0.42; 1.23]	465	24	4.7%
Random effects model			0.63 [0.52; 0.76]			56.0%
Heterogeneity: $I^2 = 43\%$, $\tau^2 = 0.0372$, $p = 0.06$						
**Sex = Women**						
ActiFE	-0.63	0.2638	0.53 [0.32; 0.89]	132	23	4.9%
CARDIA	-2.34	0.8265	0.10 [0.02; 0.49]	314	2	0.8%
FHS	-0.41	0.3751	0.66 [0.32; 1.38]	631	11	3.1%
NES	-1.03	0.8630	0.36 [0.07; 1.94]	48	2	0.8%
JHS	-0.02	0.3910	0.98 [0.45; 2.10]	60	13	2.9%
NNPAS1	-0.05	0.4670	0.95 [0.38; 2.37]	407	8	2.2%
Tasped	-0.40	0.3155	0.67 [0.36; 1.24]	341	17	4.0%
WHS	-0.58	0.1236	0.56 [0.44; 0.71]	4185	103	8.8%
NHANES	-0.87	0.1855	0.42 [0.29; 0.60]	297	45	6.9%
NAVIGATOR	0.13	0.1931	1.14 [0.78; 1.67]	925	56	6.7%
HAI	-0.65	0.3975	0.52 [0.24; 1.13]	484	12	2.9%
Random effects model			0.62 [0.47; 0.81]			44.0%
Heterogeneity: $I^2 = 57\%$, $\tau^2 = 0.0983$, $p < 0.01$						
**Random effects model**			**0.62 [0.54; 0.73]**			**100.0%**
Heterogeneity: $I^2 = 49\%$, $\tau^2 = 0.0531$, $p < 0.01$						
Test for subgroup differences: $\chi^2_1 = 0.01$, df = 1 ($p = 0.93$)						

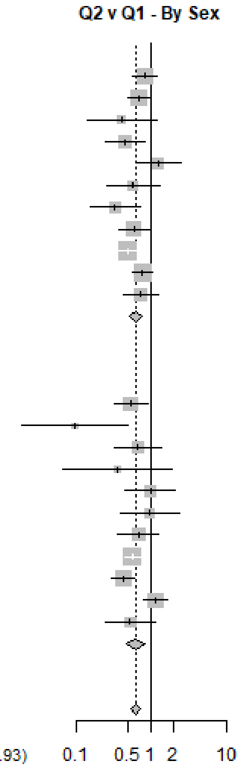

Q3 v Q1 - By Sex

Study	TE	seTE	HR(95%CI)	n	deaths	weight
**Sex = Men**						
ActiFE	-0.19	0.1986	0.83 [0.56; 1.23]	179	49	7.3%
BRHS	-0.65	0.2011	0.52 [0.35; 0.77]	350	42	7.2%
CARDIA	-0.72	0.4792	0.49 [0.19; 1.25]	226	9	2.4%
FHS	-0.52	0.3038	0.59 [0.33; 1.08]	524	17	4.6%
NES	0.34	0.3544	1.41 [0.70; 2.82]	51	18	3.8%
JHS	-0.92	0.4798	0.40 [0.16; 1.02]	40	7	2.4%
NNPAS1	-1.20	0.3877	0.30 [0.14; 0.64]	354	9	3.3%
Tasped	-0.56	0.2621	0.57 [0.34; 0.95]	312	23	5.5%
NHANES	-0.97	0.1579	0.38 [0.28; 0.52]	299	35	8.7%
NAVIGATOR	-0.62	0.1768	0.54 [0.38; 0.76]	894	47	8.0%
HAI	-0.73	0.3139	0.48 [0.26; 0.89]	465	16	4.4%
Random effects model			0.55 [0.44; 0.68]			57.8%
Heterogeneity: $I^2 = 50\%$, $\tau^2 = 0.0639$, $p = 0.03$						
**Sex = Women**						
ActiFE	-1.11	0.3342	0.33 [0.17; 0.64]	133	12	4.1%
CARDIA	-0.04	0.4934	0.96 [0.36; 2.52]	302	8	2.3%
FHS	-2.00	0.7533	0.14 [0.03; 0.60]	613	2	1.1%
NES	0.30	0.5984	1.35 [0.42; 4.35]	53	7	1.6%
JHS	-0.59	0.4354	0.56 [0.24; 1.31]	60	9	2.8%
NNPAS1	-0.14	0.5253	0.87 [0.31; 2.44]	407	6	2.0%
Tasped	-0.26	0.3470	0.77 [0.39; 1.52]	332	14	3.9%
WHS	-0.74	0.1451	0.48 [0.36; 0.63]	4186	77	9.2%
NHANES	-1.08	0.2456	0.34 [0.21; 0.55]	298	23	6.0%
NAVIGATOR	-0.48	0.2421	0.62 [0.39; 1.00]	925	28	6.1%
HAI	-0.54	0.3889	0.58 [0.27; 1.24]	484	15	3.3%
Random effects model			0.53 [0.41; 0.68]			42.2%
Heterogeneity: $I^2 = 34\%$, $\tau^2 = 0.0537$, $p = 0.12$						
**Random effects model**			**0.54 [0.46; 0.63]**			**100.0%**
Heterogeneity: $I^2 = 41\%$, $\tau^2 = 0.0518$, $p = 0.03$						
Test for subgroup differences: $\chi^2_1 = 0.05$, df = 1 ($p = 0.82$)						

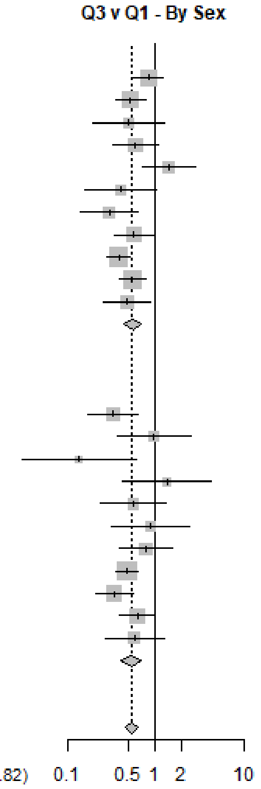

Figure S3a cont: Association of Steps/Day with All-Cause Mortality Stratified by Sex – Forest Plots

Study	TE	seTE	HR(95%CI)	n	deaths	weight
Sex = Men						
ActiFE	-0.21	0.2168	0.81 [0.53; 1.24]	178	38	7.3%
BRHS	-1.02	0.2559	0.36 [0.22; 0.59]	352	24	6.4%
CARDIA	-0.49	0.4530	0.62 [0.25; 1.49]	264	12	3.4%
FHS	-0.39	0.3141	0.67 [0.36; 1.25]	622	18	5.3%
NES	-0.71	0.4399	0.49 [0.21; 1.16]	63	8	3.6%
JHS	-1.15	0.5256	0.32 [0.11; 0.89]	52	6	2.8%
NNPAS1	-1.11	0.4072	0.33 [0.15; 0.73]	354	10	4.0%
Tasped	-0.69	0.3127	0.50 [0.27; 0.92]	295	17	5.4%
NHANES	-0.82	0.1768	0.44 [0.31; 0.62]	298	25	8.2%
NAVIGATOR	-0.20	0.1746	0.82 [0.58; 1.15]	893	58	8.2%
HAI	-1.11	0.3728	0.33 [0.16; 0.69]	465	10	4.4%
Random effects model			0.52 [0.41; 0.66]			58.9%
Heterogeneity: $I^2 = 44\%$, $\tau^2 = 0.0624$, $p = 0.06$						
Sex = Women						
ActiFE	-1.27	0.3902	0.28 [0.13; 0.60]	132	9	4.2%
CARDIA	-1.12	0.6420	0.33 [0.09; 1.15]	263	4	2.0%
FHS	-0.55	0.5398	0.58 [0.20; 1.67]	515	5	2.7%
NES	-0.85	0.8808	0.43 [0.08; 2.41]	41	2	1.2%
JHS	-0.99	0.5904	0.37 [0.12; 1.18]	49	4	2.3%
NNPAS1	-0.29	0.5820	0.75 [0.24; 2.35]	407	5	2.4%
Tasped	0.04	0.3647	1.04 [0.51; 2.13]	349	14	4.5%
WHS	-1.12	0.1812	0.33 [0.23; 0.47]	4185	49	8.1%
NHANES	-0.92	0.2689	0.40 [0.24; 0.68]	297	22	6.2%
NAVIGATOR	-0.48	0.2594	0.62 [0.37; 1.03]	924	25	6.4%
HAI	-3.22	0.8920	0.04 [0.01; 0.23]	483	1	1.2%
Random effects model			0.43 [0.31; 0.61]			41.1%
Heterogeneity: $I^2 = 50\%$, $\tau^2 = 0.1409$, $p = 0.03$						
**Random effects model**			**0.48 [0.39; 0.59]**			**100.0%**
Heterogeneity: $I^2 = 50\%$, $\tau^2 = 0.0956$, $p < 0.01$						
Test for subgroup differences: $\chi^2_1 = 0.82$, df = 1 ($p = 0.37$)						

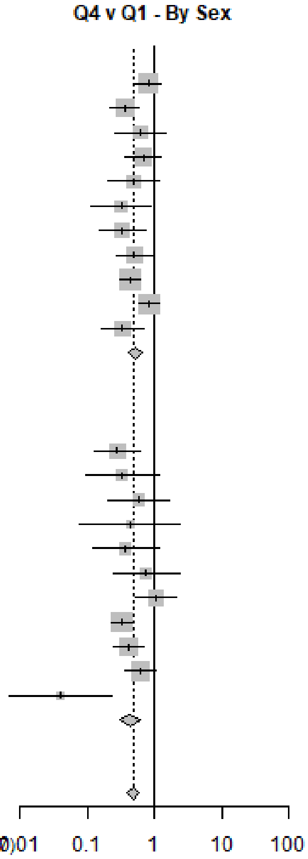

TE = treatment effect (log hazard ratio); seTE = standard error of treatment estimate; Q = Quartile; HR (95% CI) = Hazard Ratio and 95% Confidence Intervals.
Models adjusted for age, accelerometer wear time, race/ethnicity (if applicable), education or occupation, BMI, and study-specific variables for lifestyle, chronic conditions or risk factors, and general health status (see supplement 1a).

Figure S3b
Median Steps/d for quartiles by Sex among studies included in Sex Stratified Spline analysis

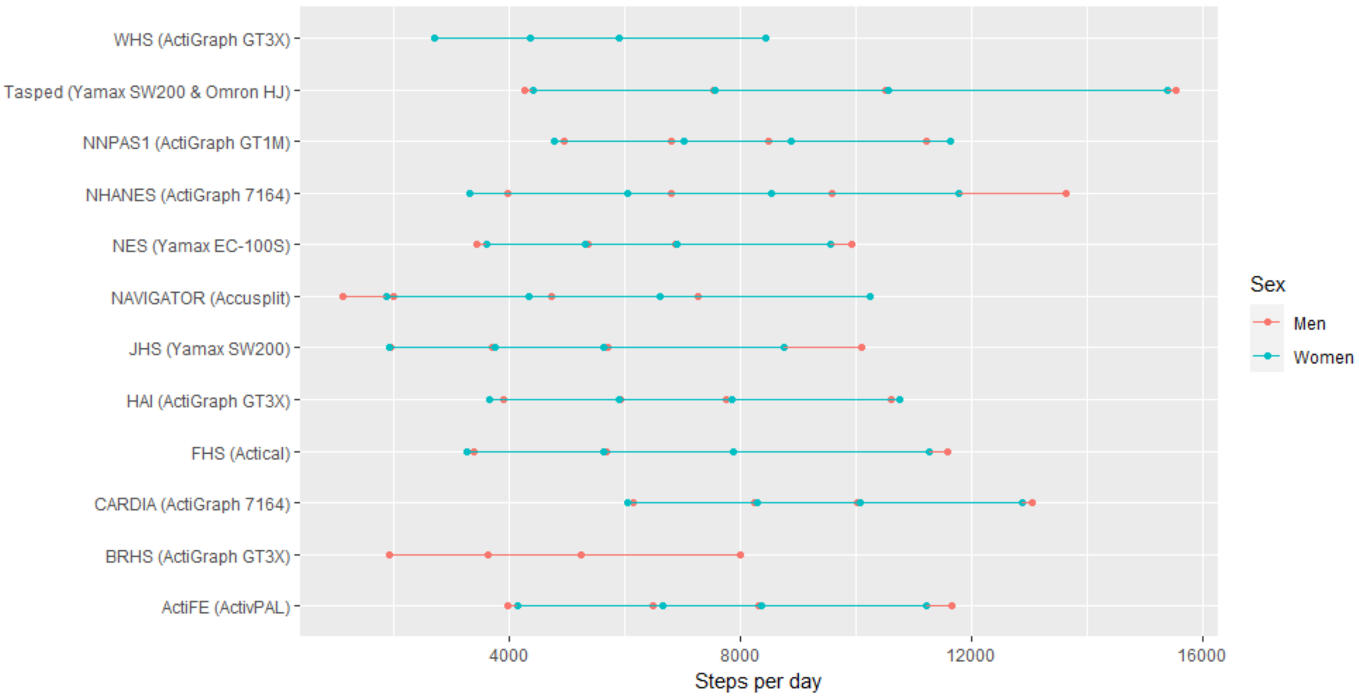

Figure S3c
Sex Stratified Restricted Cubic Spline – Final adjusted Model

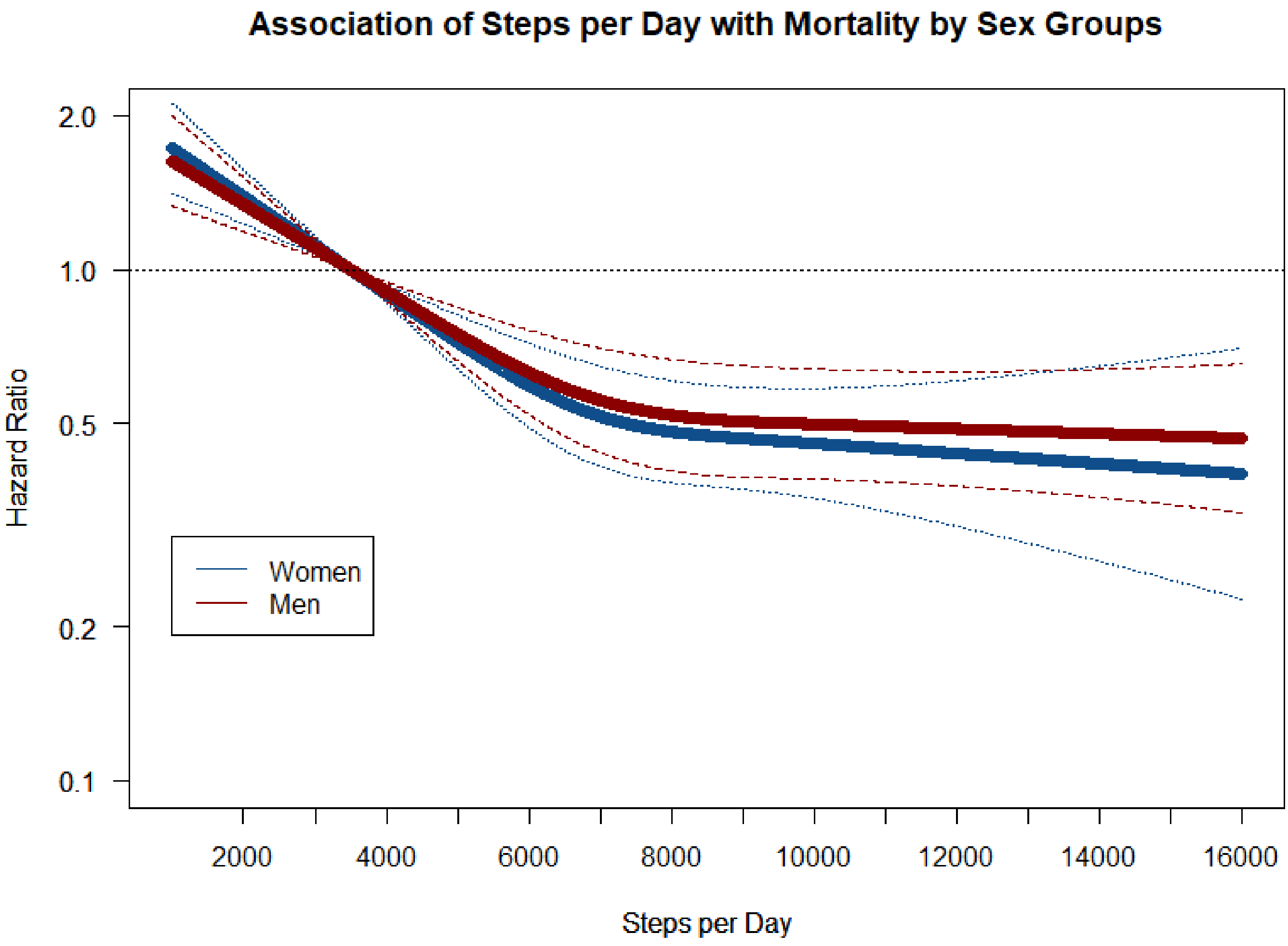

Hazard Ratio and 95% Confidence Intervals [HR (95% CI)] adjusted for age, accelerometer wear time, race/ethnicity/ethnicity (if applicable), sex (if applicable), education or occupation, BMI, and study-specific variables for lifestyle, chronic conditions or risk factors, and general health status (see supplement 1a). Reference set 3,500 steps/d (median of the lowest quartile median). P-value = 0.1203 for interaction by sex

Figure S4a: Peak 30 Minute Intensity – Forest Plots

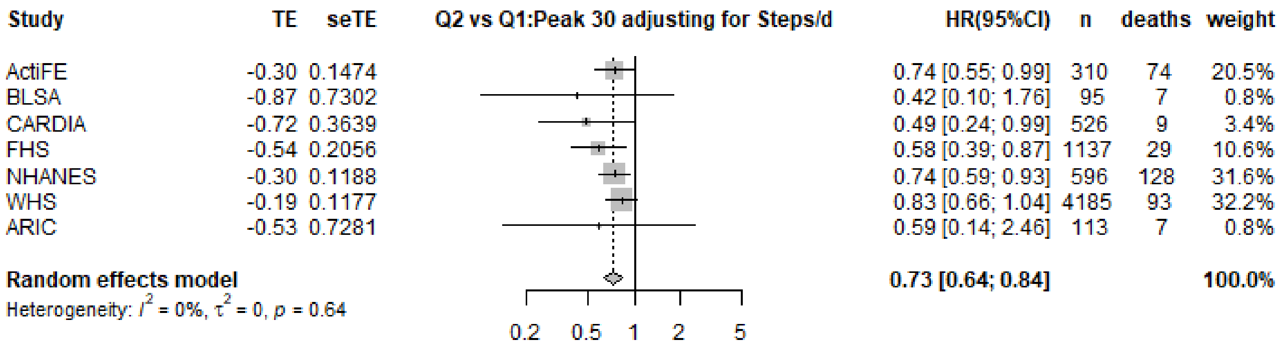

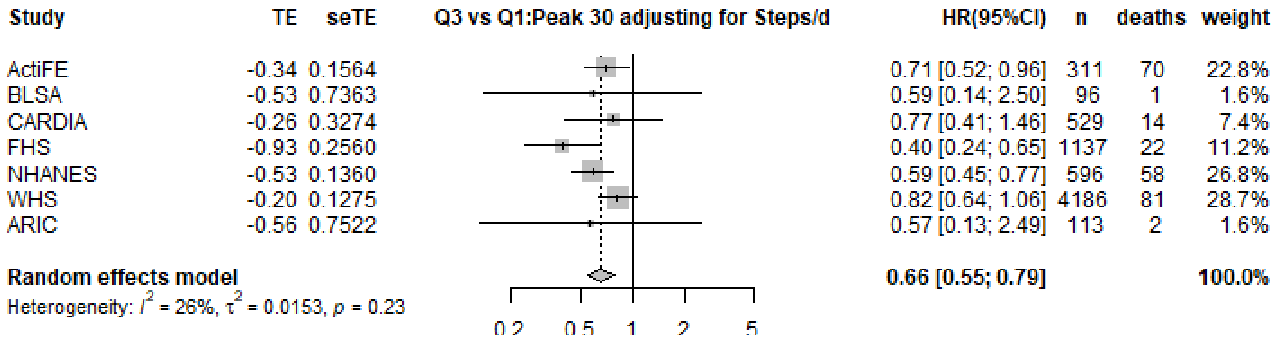

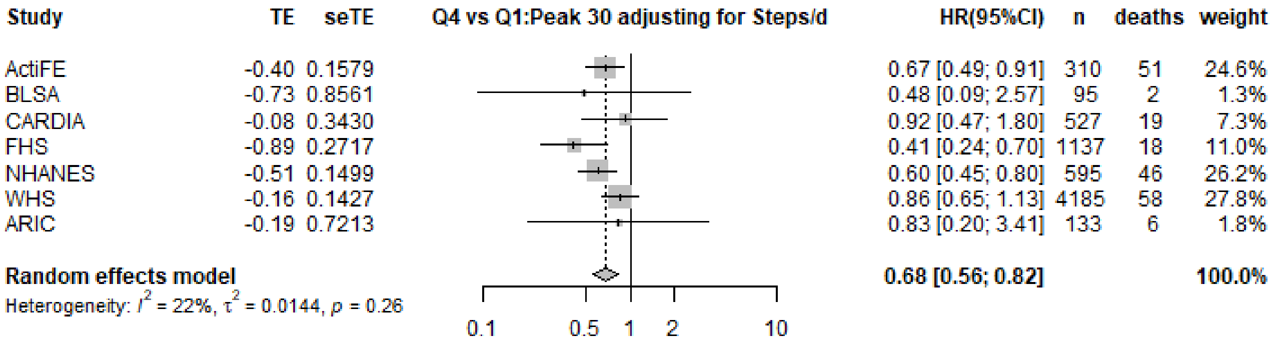

Peak 30 minute quantified as the highest steps/min observed in any 30 minutes, not necessarily consecutive, throughout a sing le day, and averaged across days.
TE = treatment effect (log hazard ratio); seTE = standard error of treatment estimate; Q = Quartile; HR (95% CI) = Hazard Ratio and 95% Confidence Intervals. Models adjusted for age, accelerometer wear time, race/ethnicity (if applicable), sex (if applicable), education or occupation, BMI, and study-specific variables for lifestyle, chronic conditions or risk factors, and general health status (see supplement 1a), plus steps/d using the residual method.

Figure S4b: Peak 30 Intensity Medians by Quartile

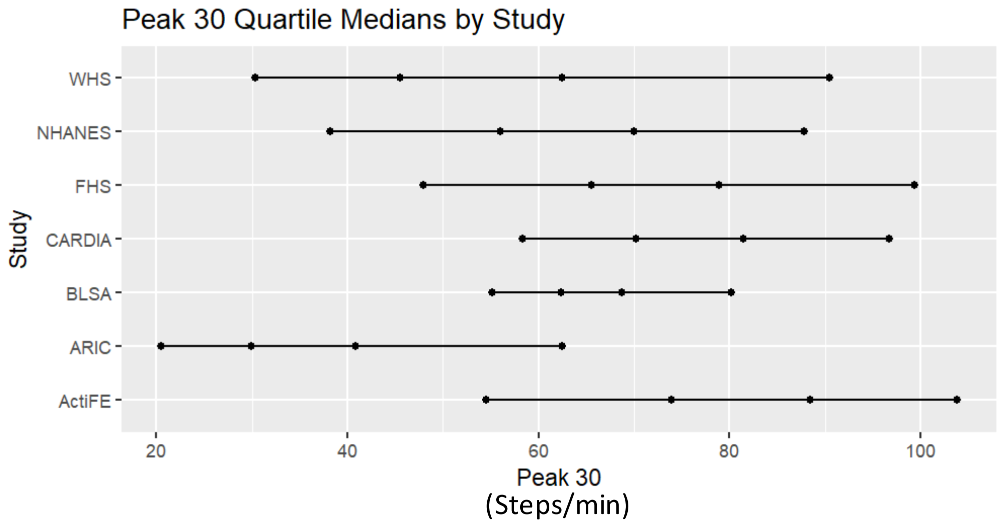

Figure S5a: Peak 60 Minute Intensity – Forest Plots

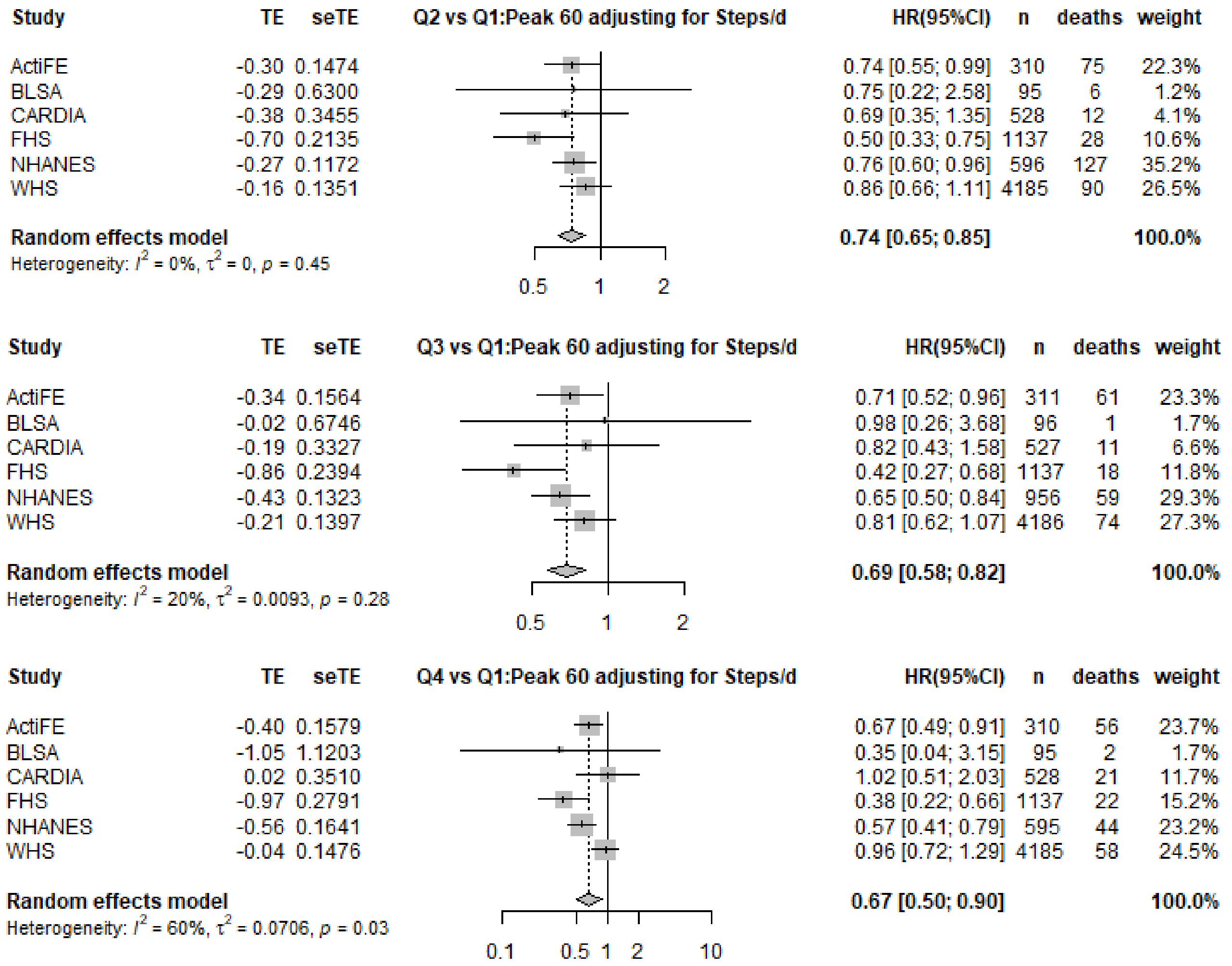

Peak 60 minute quantified as the highest steps/min observed in any 60 minutes, not necessarily consecutive, throughout a single day, and averaged across days.

TE = treatment effect (log hazard ratio); seTE = standard error of treatment estimate; Q = Quartile; HR (95% CI) = Hazard Ratio and 95% Confidence Intervals. Models adjusted for age, accelerometer wear time, race/ethnicity (if applicable), sex (if applicable), education or occupation, BMI, and study-specific variables for lifestyle, chronic conditions or risk factors, and general health status (see supplement 1a), plus steps/d using the residual method.

Figure S5b: Peak 60 Minute Intensity Medians by Quartile

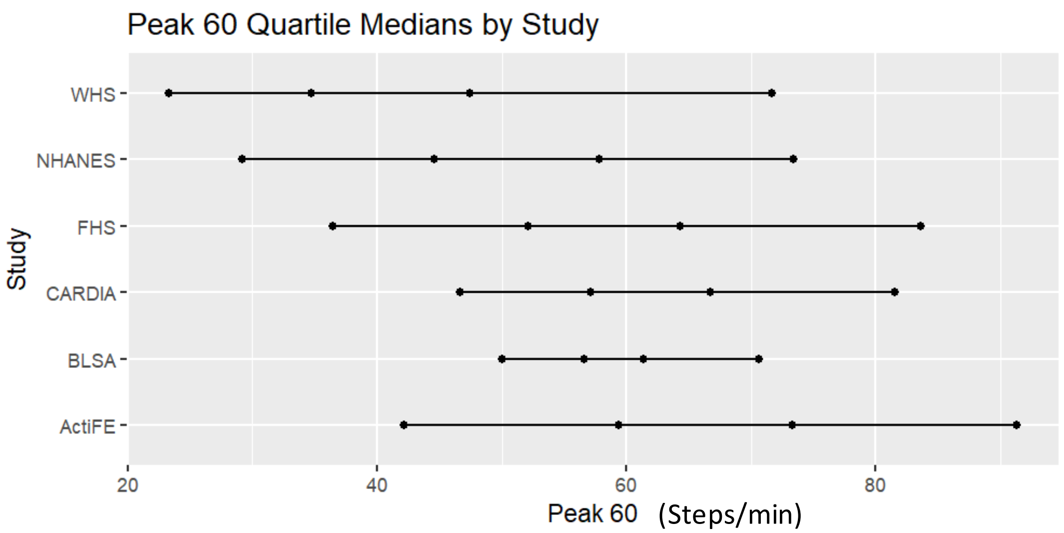

Figure S6a: Time Spent at ≥40 steps/min Intensity – Forest Plots

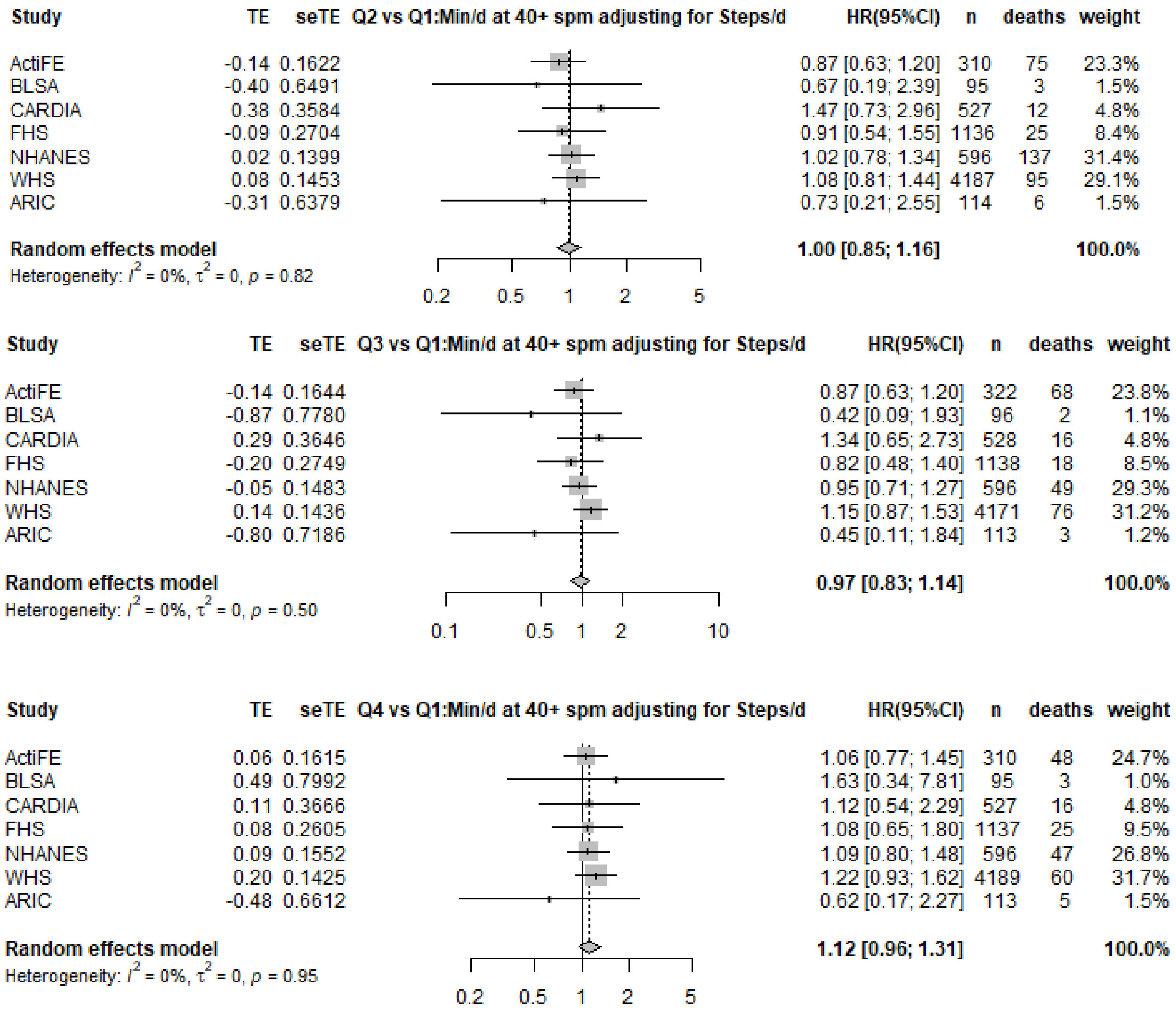

Time spent at ≥40 steps/min is quantified as the *average duration (minutes per day) of steps accumulated at ≥40 steps/min, considered intentional walking*

TE = treatment effect (log hazard ratio); seTE = standard error of treatment estimate; Q = Quartile; HR (95% CI) = Hazard Ratio and 95% Confidence Intervals. Models adjusted for age, accelerometer wear time, race/ethnicity (if applicable), sex (if applicable), education or occupation, BMI, and study-specific variables for lifestyle, chronic conditions or risk factors, and general health status (see supplement 1a), plus steps/d using the residual method.

Figure S6b: Time Spent at ≥40 steps/min Intensity Medians by Quartile

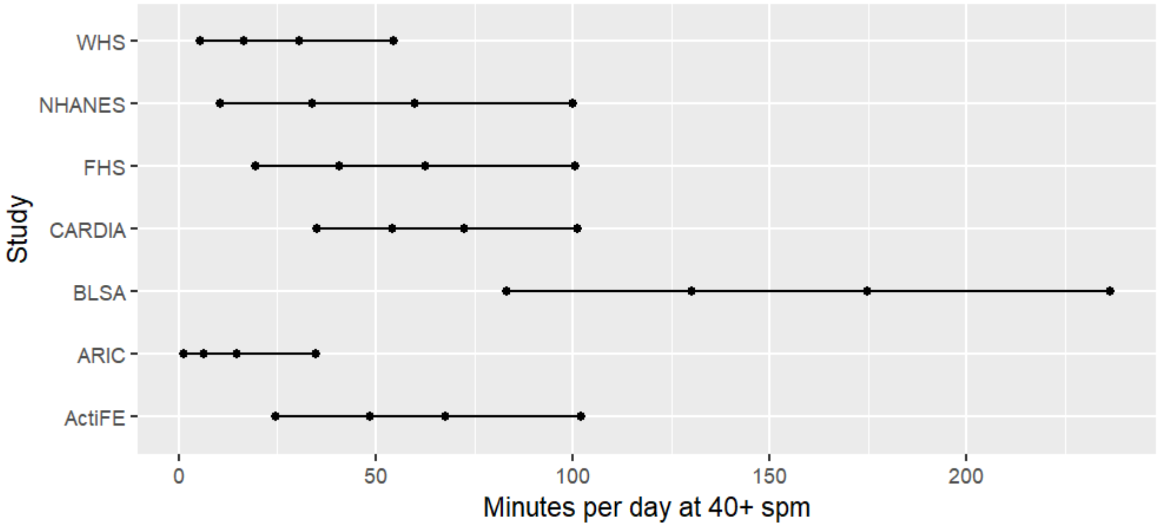

Figure S7a: Time Spent at ≥100 steps/min Intensity – Forest Plots

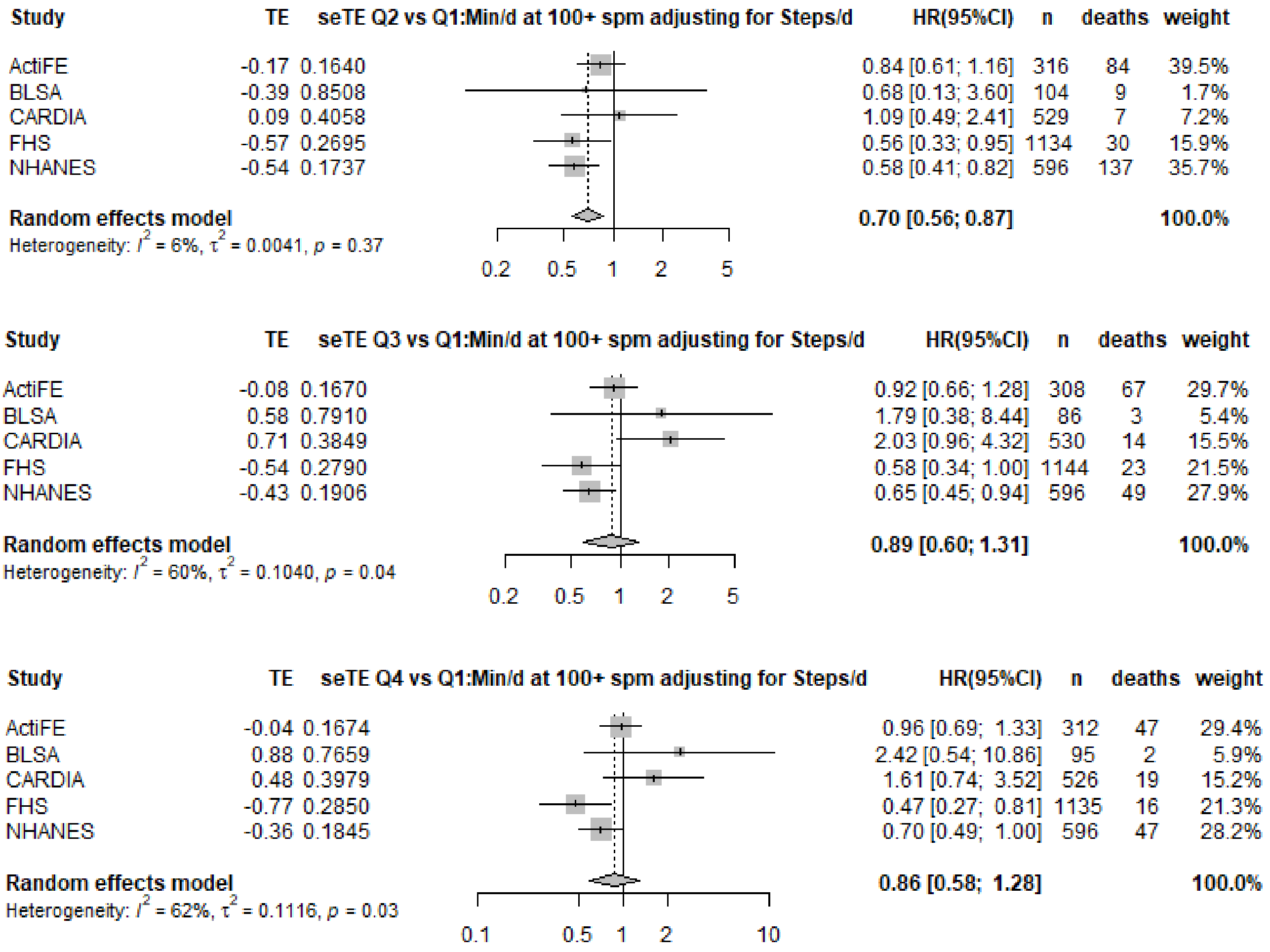

Time spent at ≥100 steps/min is quantified as the *average duration (minutes per day) of steps accumulated at ≥100 steps/min, considered moderate intensity walking*

TE = treatment effect (log hazard ratio); seTE = stardard error of treatment estimate; Q = Quartile; HR (95% CI) = Hazard Ratio and 95% Confidence Intervals. Models adjusted for age, accelerometer wear time, race/ethnicity (if applicable), sex (if applicable), education or occupation, BMI, and study-specific variables for lifestyle, chronic conditions or risk factors, and general health status (see supplement 1a), plus steps/d using the residual method.

Figure S7b: Time Spent at ≥100 steps/min Intensity Medians by Quartile

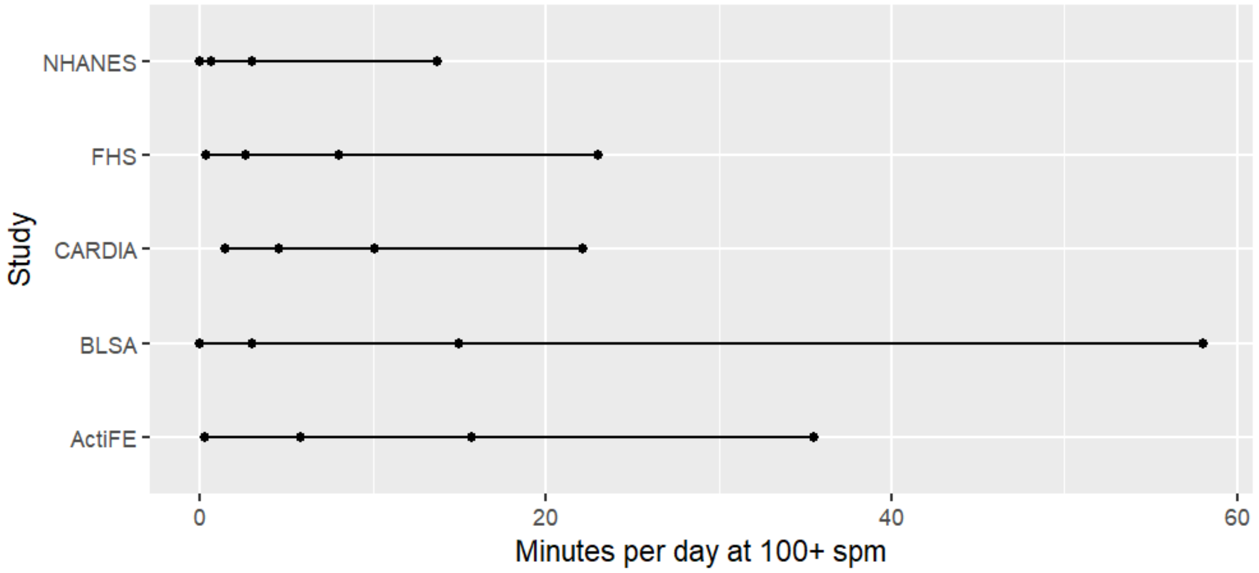

Table S4: Sensitivity – Removing Deaths in First 2 Years

Summary Results for Association of Steps per Day with Mortality - Removing Deaths in First 2 Years of Follow-up				
Steps/d Quartile	Total	deaths	HR [95% CI]	HR [95% CI]
			Model 1	Model 2
Q1	11252	1133	ref	ref
Q2	11368	549	0.58 [0.49; 0.70]	0.67 [0.58; 0.78]
Q3	11385	394	0.50 [0.42; 0.60]	0.58 [0.50; 0.68]
Q4	11428	314	0.42 [0.34; 0.52]	0.52 [0.43; 0.65]

k=13 studies (exclude CPS-3 and ARIC because of shorter follow-up and smaller no. of deaths)

HR [95% CI] = Hazard Ratio and 95% Confidence Intervals.
Model 1: adjusted for age, and sex (if applicable)
Model 2: adjusted for age, sex, (if applicable) accelerometer wear time, race/ethnicity (if applicable), education or occupation, BMI, and study-specific variables for lifestyle, chronic conditions or risk factors, and general health status (see supplement 1a).

Figure S8. Sensitivity – Removing Deaths in First 2 Years, forest plots

Q2 v Q1: Exclude first 2 yr deaths

Study	TE	seTE	HR(95%CI)	n	deaths	weight
ActiFE	-0.11	0.1567	0.90 [0.66; 1.22]	299	74	11.7%
BRHS	-0.30	0.1988	0.74 [0.50; 1.09]	338	49	9.0%
CARDIA	-1.39	0.4321	0.25 [0.11; 0.58]	528	7	2.7%
FHS	-0.62	0.2734	0.54 [0.32; 0.92]	1126	19	5.8%
NES	-0.01	0.3735	0.99 [0.48; 2.06]	98	16	3.5%
NNPAS1	-0.56	0.2932	0.57 [0.32; 1.01]	758	17	5.2%
Tasped	-0.39	0.1977	0.68 [0.46; 1.00]	641	41	9.1%
WHS	-0.61	0.1499	0.54 [0.40; 0.73]	4131	69	12.3%
JHS	-0.23	0.2819	0.79 [0.46; 1.38]	100	25	5.6%
NHANES	-0.54	0.1269	0.58 [0.45; 0.74]	575	98	14.2%
BLSA	-1.08	0.7478	0.34 [0.08; 1.47]	71	4	1.0%
NAVIGATOR	-0.15	0.1326	0.86 [0.66; 1.12]	1764	105	13.7%
HAI	-0.45	0.2641	0.64 [0.38; 1.07]	939	25	6.1%
**Random effects model**			**0.67 [0.58; 0.78]**			**100.0%**

Heterogeneity: $I^2 = 37\%$, $\tau^2 = 0.0250$, $p = 0.09$

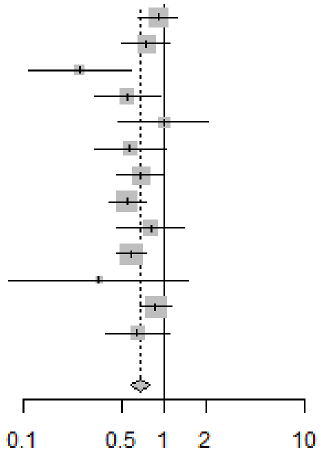

Q3 v Q1: Exclude first 2 yr deaths

Study	TE	seTE	HR(95%CI)	n	deaths	weight
ActiFE	-0.26	0.1768	0.77 [0.54; 1.09]	300	56	11.8%
BRHS	-0.67	0.2291	0.51 [0.33; 0.80]	339	31	8.6%
CARDIA	-0.61	0.3295	0.54 [0.28; 1.04]	527	16	5.0%
FHS	-0.63	0.3008	0.53 [0.29; 0.96]	1134	16	5.8%
NES	0.45	0.3438	1.58 [0.80; 3.09]	99	22	4.7%
NNPAS1	-0.67	0.3117	0.51 [0.28; 0.94]	758	15	5.5%
Tasped	-0.33	0.2192	0.72 [0.47; 1.11]	641	34	9.1%
WHS	-0.86	0.1811	0.42 [0.30; 0.60]	4139	47	11.5%
JHS	-0.71	0.3235	0.49 [0.26; 0.93]	100	16	5.2%
NHANES	-0.73	0.1730	0.48 [0.34; 0.67]	575	53	12.1%
BLSA	-1.11	0.8747	0.33 [0.06; 1.83]	71	2	0.9%
NAVIGATOR	-0.51	0.1588	0.60 [0.44; 0.82]	1764	63	13.2%
HAI	-0.56	0.2777	0.57 [0.33; 0.98]	938	23	6.6%
**Random effects model**			**0.58 [0.50; 0.68]**			**100.0%**

Heterogeneity: $I^2 = 32\%$, $\tau^2 = 0.0261$, $p = 0.13$

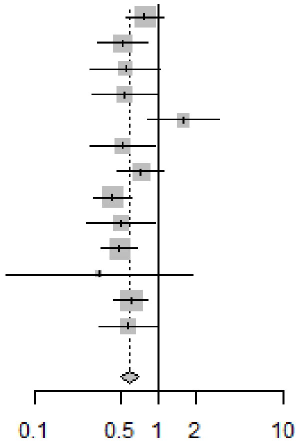

Q4 v Q1: Exclude first 2 yr deaths

Study	TE	seTE	HR(95%CI)	n	deaths	weight
ActiFE	-0.29	0.1961	0.75 [0.51; 1.10]	299	44	12.2%
BRHS	-0.99	0.2921	0.37 [0.21; 0.66]	346	18	7.4%
CARDIA	-0.79	0.3427	0.45 [0.23; 0.88]	526	15	5.8%
FHS	-0.37	0.3048	0.69 [0.38; 1.25]	1127	18	6.9%
NES	-0.65	0.4289	0.52 [0.23; 1.21]	102	9	4.0%
NNPAS1	-0.84	0.3626	0.43 [0.21; 0.88]	759	12	5.3%
Tasped	-0.31	0.2538	0.73 [0.44; 1.20]	649	27	9.0%
WHS	-0.86	0.2036	0.42 [0.29; 0.63]	4170	43	11.7%
JHS	-0.99	0.3907	0.37 [0.17; 0.80]	101	10	4.7%
NHANES	-0.71	0.1878	0.49 [0.34; 0.71]	575	43	12.7%
BLSA	-0.43	0.8568	0.65 [0.12; 3.49]	71	3	1.1%
NAVIGATOR	-0.39	0.1610	0.68 [0.50; 0.93]	1764	65	14.7%
HAI	-1.61	0.4106	0.20 [0.09; 0.45]	939	7	4.4%
**Random effects model**			**0.52 [0.43; 0.63]**			**100.0%**

Heterogeneity: $I^2 = 32\%$, $\tau^2 = 0.0340$, $p = 0.12$

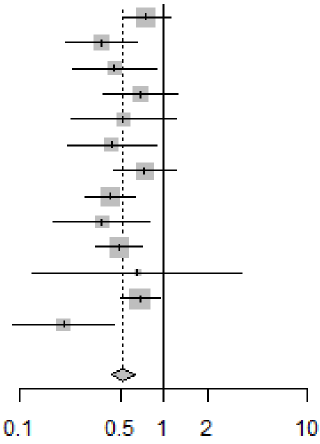

TE = treatment effect (log hazard ratio); seTE = standard error of treatment estimate; Q = Quartile; HR (95% CI) = Hazard Ratio and 95% Confidence Intervals. Models adjusted for age, accelerometer wear time, race/ethnicity (if applicable), sex (if applicable), education or occupation, BMI, and study-specific variables for lifestyle, chronic conditions or risk factors, and general health status (see supplement 1a)

29

Figure S9. Stratified by < or > 6 Years of Study Follow-up

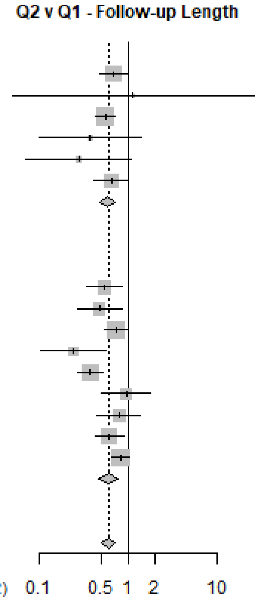

Study	TE	seTE	HR(95%CI)	n	deaths	weight
Follow = <6 years						
BRHS	-0.39	0.1768	0.68 [0.48; 0.96]	349	60	9.4%
CPS-3	0.14	1.5902	1.15 [0.05; 25.96]	180	2	0.3%
WHS	-0.58	0.1236	0.56 [0.44; 0.71]	4185	103	11.8%
BLSA	-0.97	0.6787	0.38 [0.10; 1.44]	95	5	1.4%
ARIC	-1.27	0.6980	0.28 [0.07; 1.10]	113	3	1.4%
HAI	-0.43	0.2213	0.65 [0.42; 1.00]	948	36	7.6%
Random effects model			0.59 [0.50; 0.71]			31.8%
Heterogeneity: $I^2 = 0\%$, $\tau^2 = 0$, $p = 0.73$						
Follow = >6 years						
FHS	-0.61	0.2391	0.55 [0.34; 0.87]	1137	25	7.0%
NNPAS1	-0.73	0.2926	0.48 [0.27; 0.85]	761	17	5.5%
ActiFE	-0.31	0.1447	0.73 [0.55; 0.97]	310	84	10.8%
CARDIA	-1.41	0.4311	0.24 [0.11; 0.57]	528	7	3.1%
NHANES	-0.97	0.1579	0.38 [0.28; 0.52]	596	112	10.2%
NES	-0.04	0.3228	0.96 [0.51; 1.82]	104	20	4.8%
JHS	-0.23	0.2819	0.79 [0.46; 1.38]	100	25	5.8%
Tasped	-0.48	0.1884	0.62 [0.43; 0.90]	644	44	8.9%
NAVIGATOR	-0.17	0.1170	0.84 [0.67; 1.06]	1827	133	12.1%
Random effects model			0.60 [0.47; 0.77]			68.2%
Heterogeneity: $I^2 = 69\%$, $\tau^2 = 0.0877$, $p < 0.01$						
**Random effects model**			**0.60 [0.51; 0.71]**			**100.0%**

Heterogeneity: $I^2 = 52\%$, $\tau^2 = 0.0462$, $p = 0.01$
Test for subgroup differences: $\chi^2_1 = 0.01$, df = 1 ($p = 0.92$)

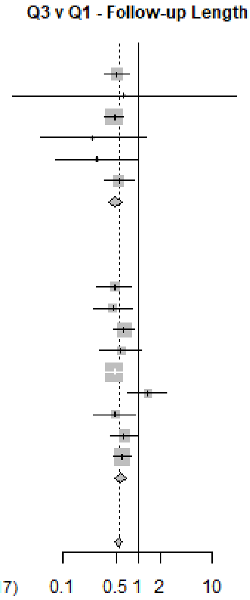

Study	TE	seTE	HR(95%CI)	n	deaths	weight
Follow = <6 years						
BRHS	-0.65	0.2011	0.52 [0.35; 0.77]	350	42	8.1%
CPS-3	-0.43	1.7569	0.65 [0.02; 20.34]	180	1	0.1%
WHS	-0.74	0.1451	0.48 [0.36; 0.63]	4186	77	13.8%
BLSA	-1.39	0.8312	0.25 [0.05; 1.27]	96	2	0.5%
ARIC	-1.27	0.6519	0.28 [0.08; 1.00]	113	3	0.9%
HAI	-0.60	0.2352	0.55 [0.35; 0.87]	950	32	6.1%
Random effects model			0.49 [0.40; 0.60]			29.6%
Heterogeneity: $I^2 = 0\%$, $\tau^2 = 0$, $p = 0.88$						
Follow = >6 years						
FHS	-0.74	0.2732	0.48 [0.28; 0.82]	1137	19	4.7%
NNPAS1	-0.78	0.2992	0.46 [0.26; 0.83]	761	16	4.0%
ActiFE	-0.43	0.1657	0.65 [0.47; 0.90]	310	84	11.2%
CARDIA	-0.53	0.3230	0.59 [0.31; 1.10]	528	17	3.4%
NHANES	-0.73	0.1207	0.48 [0.38; 0.61]	596	58	18.0%
NES	0.29	0.3034	1.34 [0.74; 2.43]	104	25	3.9%
JHS	-0.71	0.3235	0.49 [0.26; 0.93]	100	16	3.4%
Tasped	-0.43	0.2101	0.65 [0.43; 0.98]	644	37	7.5%
NAVIGATOR	-0.49	0.1412	0.61 [0.46; 0.80]	1822	82	14.4%
Random effects model			0.59 [0.50; 0.70]			70.4%
Heterogeneity: $I^2 = 35\%$, $\tau^2 = 0.0226$, $p = 0.14$						
**Random effects model**			**0.55 [0.49; 0.62]**			**100.0%**

Heterogeneity: $I^2 = 12\%$, $\tau^2 = 0.0065$, $p = 0.32$
Test for subgroup differences: $\chi^2_1 = 1.86$, df = 1 ($p = 0.17$)

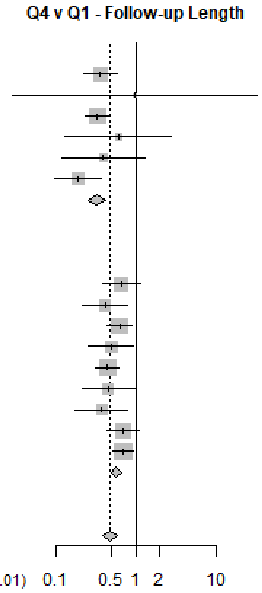

Study	TE	seTE	HR(95%CI)	n	deaths	weight
Follow = <6 years						
BRHS	-1.02	0.2559	0.36 [0.22; 0.59]	352	24	8.0%
CPS-3	-0.04	1.8020	0.96 [0.03; 32.82]	180	1	0.3%
WHS	-1.12	0.1812	0.33 [0.23; 0.47]	4185	49	10.8%
BLSA	-0.51	0.7813	0.60 [0.13; 2.77]	95	3	1.5%
ARIC	-0.94	0.6117	0.39 [0.12; 1.29]	113	5	2.3%
HAI	-1.66	0.3472	0.19 [0.10; 0.38]	948	10	5.6%
Random effects model			0.32 [0.25; 0.41]			28.5%
Heterogeneity: $I^2 = 0\%$, $\tau^2 = 0$, $p = 0.61$						
Follow = >6 years						
FHS	-0.41	0.2694	0.66 [0.39; 1.12]	1137	23	7.6%
NNPAS1	-0.89	0.3412	0.41 [0.21; 0.80]	761	14	5.7%
ActiFE	-0.46	0.1826	0.63 [0.44; 0.90]	310	48	10.8%
CARDIA	-0.72	0.3341	0.49 [0.25; 0.94]	527	16	5.9%
NHANES	-0.82	0.1768	0.44 [0.31; 0.62]	595	53	11.0%
NES	-0.79	0.3891	0.45 [0.21; 0.97]	104	10	4.7%
JHS	-0.99	0.3907	0.37 [0.17; 0.80]	101	10	4.7%
Tasped	-0.37	0.2338	0.69 [0.44; 1.09]	644	31	8.8%
NAVIGATOR	-0.37	0.1455	0.69 [0.52; 0.92]	1807	82	12.4%
Random effects model			0.57 [0.49; 0.66]			71.5%
Heterogeneity: $I^2 = 0\%$, $\tau^2 = 0$, $p = 0.45$						
**Random effects model**			**0.47 [0.39; 0.57]**			**100.0%**

Heterogeneity: $I^2 = 47\%$, $\tau^2 = 0.0599$, $p = 0.02$
Test for subgroup differences: $\chi^2_1 = 14.78$, df = 1 ($p < 0.01$)

TE = treatment effect (log hazard ratio); seTE = standard error of treatment estimate; Q = Quartile; HR (95% CI) = Hazard Ratio and 95% Confidence Intervals. Models adjusted for age, accelerometer wear time, race/ethnicity (if applicable), sex (if applicable), education or occupation, BMI, and study-specific variables for lifestyle, chronic conditions or risk factors, and general health status (see supplement 1a)

Figure S10a. Forest Plots Stratified by Publication Status (Yes/No)

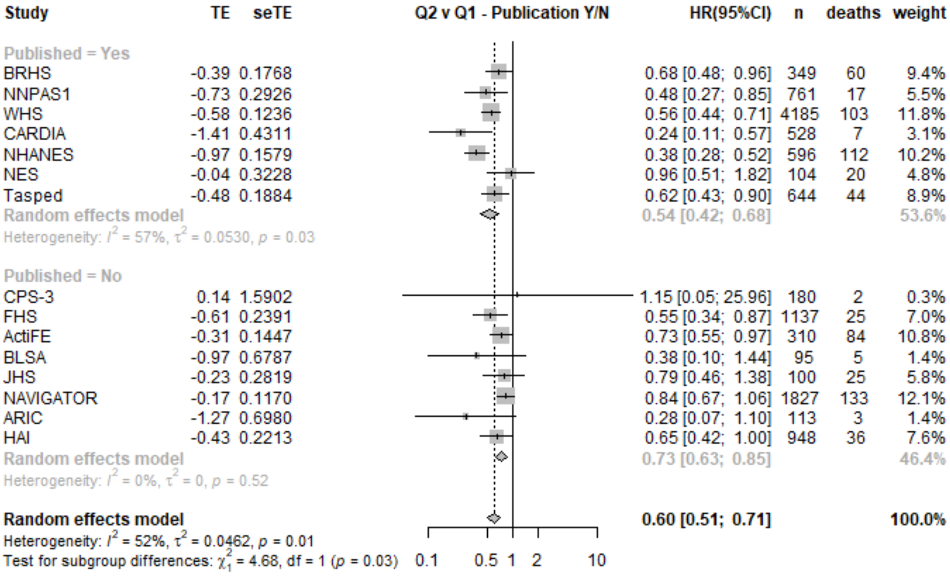

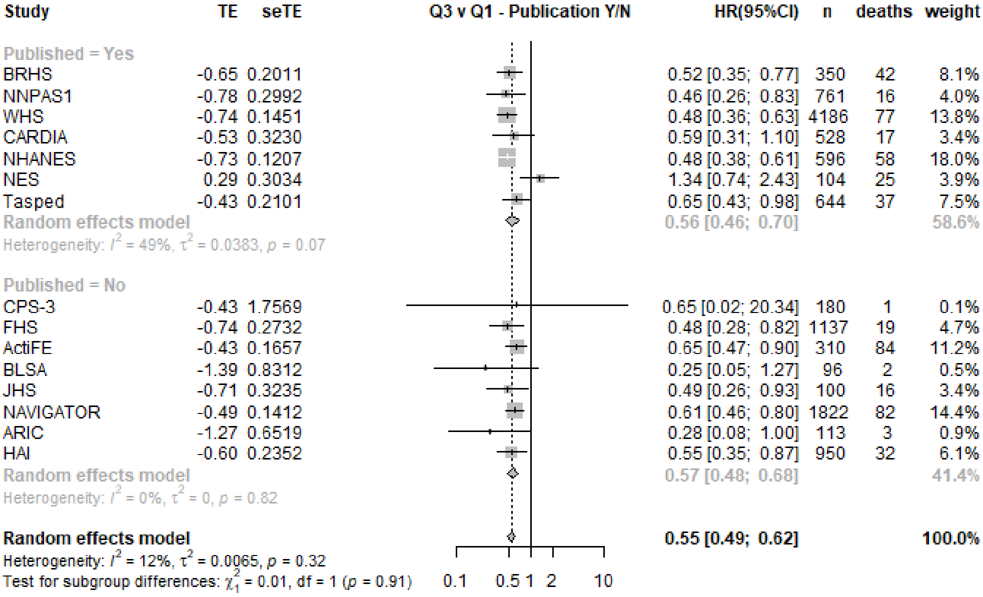

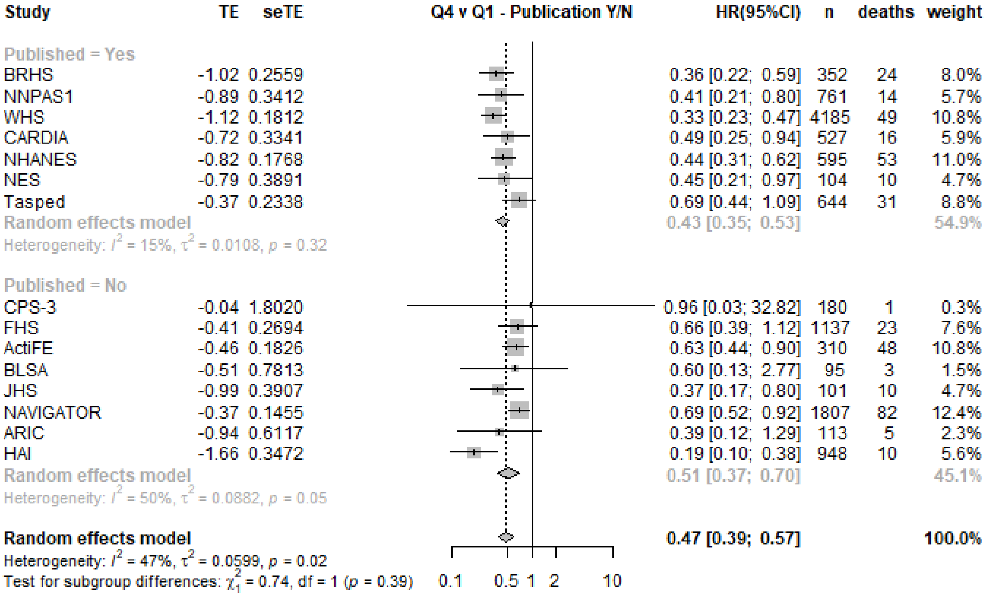

TE = treatment effect (log hazard ratio); seTE = standard error of treatment estimate; Q = Quartile; HR (95% CI) = Hazard Ratio and 95% Confidence Intervals. Models adjusted for age, accelerometer wear time, race/ethnicity (if applicable), sex (if applicable), education or occupation, BMI, and study-specific variables for lifestyle, chronic conditions or risk factors, and general health status (see supplement 1a)

Figure S10b.
Publication Status Stratified Restricted Cubic Spline – Final adjusted Model

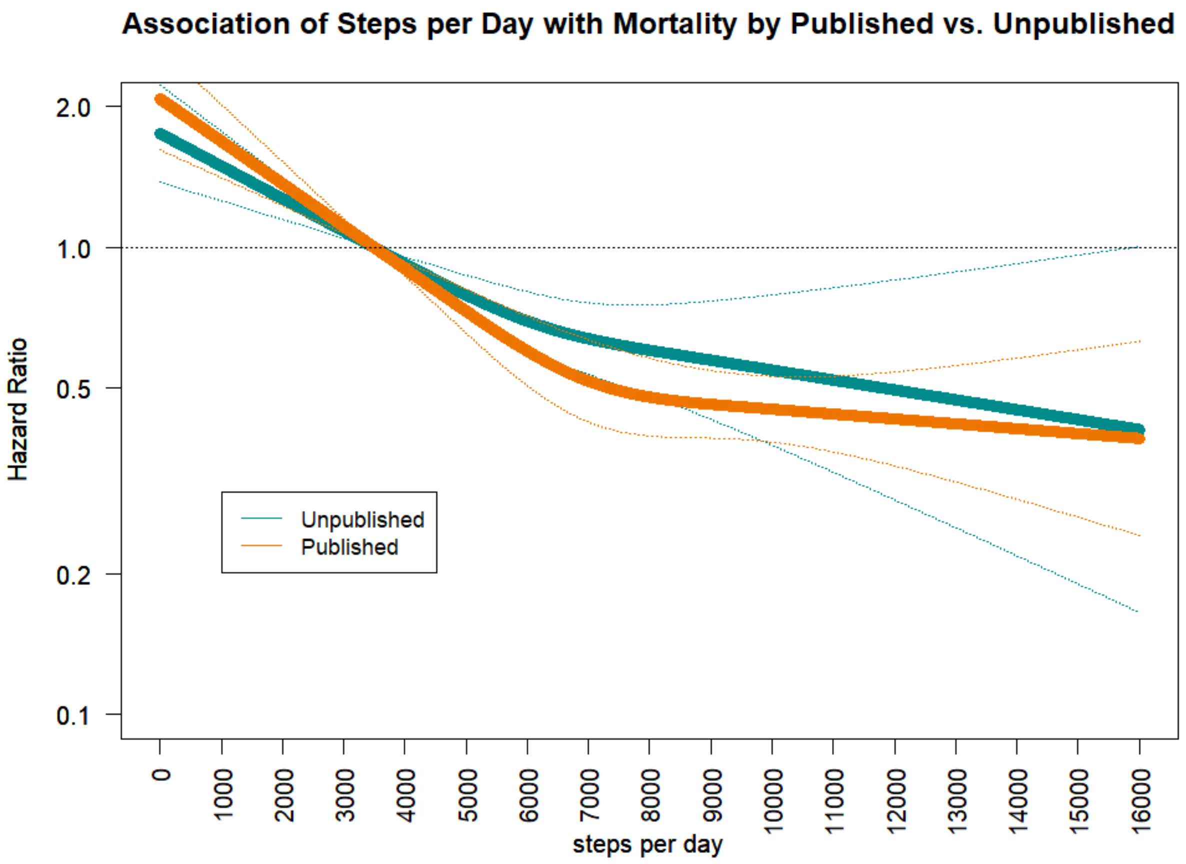

Hazard Ratio and 95% Confidence Intervals [HR (95% CI)] adjusted for age, accelerometer wear time, race/ethnicity/ethnicity (if applicable), sex (if applicable), education or occupation, BMI, and study-specific variables for lifestyle, chronic conditions or risk factors, and general health status (see supplement 1a). Reference set 3,500 steps/d (median of the lowest quartile median).

Both published and unpublished splines were significant for non-linearity. Wald tests were used to test for non-linearity by examining the null hypothesis that the regression coefficient of the spline transformation was equal to zero.

Table S5.
Leave-One-Study Out Sensitivity Analyses – Comparing Quartile 4 (most steps) vs 1 (ref: least steps)

Study left out	HR	95% CI
BRHS	0.52	(0.39; 0.69)
CPS-3	0.50	(0.38; 0.66)
FHS	0.49	(0.37; 0.65)
NNPAS1	0.51	(0.38; 0.67)
ActiFE	0.49	(0.36; 0.65)
WHS	0.53	(0.39; 0.71)
CARDIA	0.50	(0.38; 0.67)
NHANES	0.51	(0.38; 0.68)
HAI	0.50	(0.42; 0.57)
BLSA	0.50	(0.38; 0.66)
NES	0.50	(0.38; 0.67)
JHS	0.51	(0.38; 0.68)
Tasped	0.49	(0.36; 0.65)
NAVIGATOR	0.48	(0.35; 0.64)
ARIC	0.51	(0.38; 0.67)

Figure S11.
Leave-One-Device Out – Restricted Cubic Spline – Final Adjusted

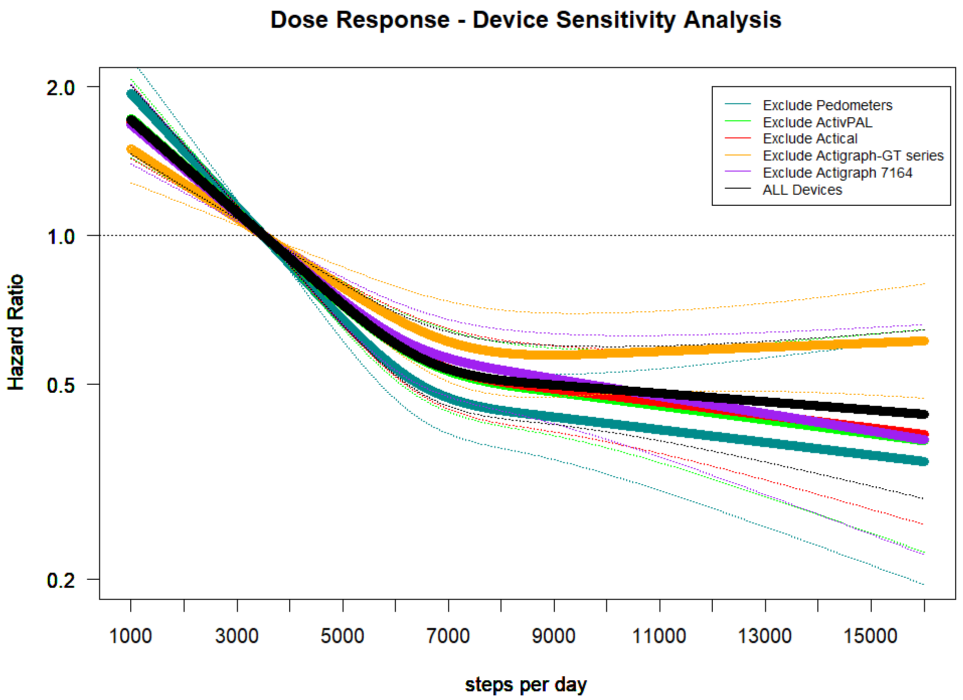

For both Figures 13a and 13b. Hazard Ratio and 95% Confidence Intervals [HR (95% CI)] adjusted for age, accelerometer wear time, race/ethnicity (if applicable), sex (if applicable), education or occupation, BMI, and study-specific variables for lifestyle, chronic conditions or risk factors, and general health status (see supplement table 1a).
Splines set at 3 knots (25, 50, and 75 percentiles), with reference at 3,500 steps/d (median of lowest quartile).

Study Overview and Statistical Analyses Instructions

Steps and Mortality Meta-analysis

TABLE OF CONTENTS

1 *Overview* ... 37
2 *Research questions* ... 37
3 *General instructions* ... 37
4 *Participant and Device Criteria* ... 38
4.1 **Participant Inclusion Criteria** ... 38
4.2 **Analytic Criteria for Accelerometer Data** ... 38
5 *Variables* ... 39
5.1 **Exposures** ... 39
5.2 **Outcomes** ... 39
5.3 **Covariates** ... 40
5.3.1 Standard covariates ... 40
5.3.2 Additional covariates ... 41
6 *Statistical Methods* ... 42
6.1 **Dose Response Association for Steps/Day with Mortality** ... 42
6.2 **Dose Response Association for Step Cadence with Mortality** ... 42
6.3 **Stratification and Sensitivity Analyses** ... 43
6.3.1 Stratification ... 43
6.3.2 Sensitivity Analyses ... 43
6.4 **Handling missing data** ... 43
7 *Table Shells for Descriptive Characteristics* ... 45
7.1 **Cohort-level Summary Characteristics** ... 45
7.2 **Baseline Descriptive Characteristics** ... 47
8 *All-Cause Mortality* ... 53
8.1 **Result tables – All-Cause Mortality** ... 53
8.1.1 Stratification and Sensitivity Analyses ... 57

Tables and Figures

7 *Table Shells for Descriptive Characteristics* ... 45
7.1 **Cohort-level Summary Characteristics** ... 45
Table 1. Study Description ... 45
Table 1 Continued. Study Description ... 46
7.2 **Baseline Descriptive Characteristics** ... 47
Table 1A. Descriptive Characteristics at Baseline for Total Sample and by Sex ... 47
Table 1Aa. Chronic Disease Definitions ... 48

Table 1B. Additional Descriptive Characteristics at Baseline for Total Sample and by Sex included in your full adjusted MODEL 3* 49
Table 1C. Step Variables at Baseline 50
Figure 1. Histogram of Distribution of Average Steps/Day per Individual at Baseline 52

All-Cause Mortality..... 53

7.3 Result tables – All-Cause Mortality 53
Table 2. Unadjusted Event Rate of All-Cause Mortality 53
Table 3: Hazard Ratios of the Association of Steps/day and All-Cause Mortality 54
Table 4. Hazard Ratios for the Association of All-Cause Mortality by Daily Stepping Intensity/Cadence 55
7.3.1 Stratification and Sensitivity Analyses 57
Supplement Table 5A. Dose Response Association of Steps/Day with All-Cause Mortality by Sex 58
Supplement Table 5B. Dose Response Association of Steps/Day with All-Cause Mortality by AGE 59
Supplement Table 5C. Dose Response Association of Steps/Day with All-Cause Mortality Excluding Individuals who Died in first 2 Years* 60

1 Overview

Steps provide an easy-to-understand metric of ambulation — an important component of physical activity for much of the population. Therefore, the measure of steps per day can be a useful tool for public health in physical activity promotion. The Physical Activity Guidelines Advisory Committee 2018 Report concluded there is limited or insufficient direct evidence to determine whether a relationship exists between step counts per day and all-cause mortality and incidence of cardiovascular disease (CVD) and type 2 diabetes. In response to the research recommendations, it is our goal to provide meaningful data on steps with prospective health outcomes.

The objective of the meta-analysis is to provide the best longitudinal evidence available on the association of steps with health outcomes, including all-cause mortality and incidence of CVD and type 2 diabetes. We plan to collaborate with cohorts that collected data on steps using device-based measures and have followed their participants for at least one or all of these health outcomes. We intend to have our final product be an impactful evidence-based analysis and publication on the dose response association of steps with these health outcomes.

2 Research questions

Primary Research Question:

1. What is the dose response association of step volume with all-cause mortality,?

Secondary Research Questions:

1. Is step cadence (intensity) related to mortality?

3 General instructions

We will set up a call prior to having you begin the analyses to answer questions and determine the best approach for completing the analyses and sharing your study's results. You can chose to provide us with the statistical output (please clearly label results with the table and figure number and titles below), or you can fill in the tables on your own. Additionally, if you may not have an analyst available to work on this project, but are still interested in participating, we would be happy to talk with you about potential ways to help you run the analyses.

For general project questions throughout the process *please do not hesitate to contact* the study coordinator, Amanda Paluch, PhD at apaluch@umass.edu..

4 PARTICIPANT AND DEVICE CRITERIA

4.1 PARTICIPANT INCLUSION CRITERIA

Adults age 18 years or older will be included in the analyses.
Compliant wear of device based measurement (see below).

4.2 ANALYTIC CRITERIA FOR ACCELEROMETER DATA

For comparability purposes across studies using different versions of accelerometers, whenever possible, please adhere to the following general criteria.

1. General settings: vertical axis (if there is a choice) using a 60 second epoch.
2. We suggest using wear time of ≥10 hours/day for ≥3 days. If you have already developed your wear time based on other criteria, this is okay, please describe your criteria below.
3. Please describe how you determined non-wear time and if applicable the non-wear classification algorithm used. We suggest using the non-wear time defined as 90-consecutive minutes of 0 counts per minute (cpm), allowing up to a 2-minute interval of non-zero cpm if the interruption is accompanied by 30 consecutive minutes of 0 cpm either up or downstream (Choi et al, *MSSE* 2011).
4. Check for outliers that may indicate implausible high or low count and step data. Please describe any criteria and reasoning for excluding outliers.

**Notes/Comments on Analytic Criteria for Accelerometer or Pedometer Data**
Please describe any unique device settings, compliance criteria, non-wear definitions, or outlier determination use specifically for your data set.

5 Variables

5.1 Exposures

Definitions of Suggested Step Variables [a]

Variable Term	Definition
Steps per day	Average number of steps recorded per day (calculated as total steps/# of compliant days)
Peak 30-min cadence	Mean steps/min from the 30 highest minutes of the day; the minutes do not need to be consecutive
Peak 60-min cadence	Mean steps/min from the 30 highest minutes of the day; the minutes do not need to be consecutive
Stepping rate of ≥40 steps/min	Corresponds to any purposeful steps taken. Will be quantified as average minutes per day and average % of time spent per day.
Stepping rate of ≥100 steps/min	Corresponds to walking at moderate intensity or greater (≥3 METs); equivalent to 2.5 mph cadence. Will be quantified as average minutes per day and average % of time spent per day.
Stepping rate of 0 steps/min	Corresponds to no stepping. Will be quantified as average minutes per day and average % of time spent per day.
Stepping rate of 1 to 39 steps/min	Corresponds to incidental or sporadic steps taken. Will be quantified as average minutes per day and average % of time spent per day.
Stepping rate of 40-99 steps/min	Corresponds to purposeful steps taken that are light intensity (<3 METs). Will be quantified as average minutes per day and average % of time spent per day.

[a] all variables based on **daily averages**

5.2 Outcomes

We will include the following outcomes:

1. All-Cause Mortality

The definitions/criteria for these outcomes will be based on standards used in your specific study.

Variable Term	Definition
All-cause mortality	

5.3 COVARIATES

5.3.1 Standard covariates

To be consistent across studies, below is a table of covariates we would like to have standardized and included in models 1 and 2 of our analyses. If you cannot quantify based on the suggested methods, please specify what you have done differently.

List of Standard Covariates and Suggested Quantification Method

Variable Term	Definition	Continuous/ Categorical	Was variable included? (Y/N)	How was your variable defined and quantified?
Age	Years	Continuous		
Wear Time	Minutes/day of wear time *if pedometer data does not have total wear time, use total number of recorded days	Continuous		
Sex	Male; Female	Categorical		
Race/ethnicity	White, Black, Hispanic, Asian, other	Categorical		
Socioeconomic Status (SES)	Use education if available (example: categorical: Less than high school; high school graduate; 4 year college graduate or above; or numerical: years of education) *However, if education is not available, please describe other SES variable used)*	Categorical		
Body Mass Index	kg/m^2	Continuous		

5.3.2 Additional covariates

We would like to have a fully adjusted model from your study as well. Based on the data available specific to your study, please run a model (Model 4) that would include as many sociodemographic, lifestyle, and health status variables you have (in a previous publication) or would plan to include in a stand-alone analysis. Please complete the table below with these variables and your definition for each.

Variable Term	Definition

6 STATISTICAL METHODS

6.1 DOSE RESPONSE ASSOCIATION FOR STEPS/DAY WITH MORTALITY

Cox Proportional Hazard **r**egression with exposures specified as quartiles and as continuous (per 1,000 steps/day). Data will be reported as hazard ratios (95% CI) and p-value for trends across quartiles of exposures. To the best of your ability please follow the following modeling strategy adjusting for the covariables measured at baseline assessment.

Modeling strategy:

Model 1: Adjust models for age and sex (if applicable)

Model 2: Model 1 + race/ethnicity, SES, and BMI (as continuous variable)

Model 3: Model 2 + average minutes/day of wear time

Model 4: Model 3 + any additional covariates specific to your study*

*Additional covariates can be any additional demographic, lifestyle, and health status variables that you would include as a final model specific to your study and/or previous publication (if applicable). These variables could be related to, but not limited to geographic region/data collection centers, smoking status, alcohol, diet, hypertension, high cholesterol, diabetes, CVD, cancer, self-rated health, functional status. Please quantify/categorize these variables as is typically done in your specific study.

6.2 DOSE RESPONSE ASSOCIATION FOR STEP CADENCE WITH MORTALITY

Cox Proportional Hazard regression with cadence specified as continuous variables and by quartiles (as specified in the Table 4 below). Data will be reported as hazard ratios (95% CI) and p for trends across quartiles of exposures. To the best of your ability please follow the following modeling strategy adjusting for the covariables measured at baseline assessment. Models 1 and 4 will be used as above replacing steps/day variable with the cadence variable.

Then a final model 5, will add steps/day using the residual method in order to account for the high correlation between steps/day and cadence. ***we can provide analytic support for the proper use of the residual method if you have not used this method before***

Modeling strategy:

Model 1: Adjust models for age and sex (if applicable)

Model 4: Model 1 + race/ethnicity, SES, BMI, average minutes/day of wear time + any additional covariates specific to your study

Model 5: Model 3 + average steps/day using the residual method

6.3 STRATIFICATION AND SENSITIVITY ANALYSES

6.3.1 Stratification

A subset of analyses will be completed with the following stratifications:

1. Sex (male/female)
2. Age (young and middle age adults <60 and older adults ≥60 years at baseline)

As in our primary analyses, we ask for these associations with steps variables as continuous and quartiles. For quartiles, please use the quartile groupings you established for the full sample (i.e. you do NOT have to create new quartiles that are sex-, race-, or age- specific)

6.3.2 Sensitivity Analyses

If applicable to your study, run an analysis that excludes those who died in first 2 years of follow-up in order to address the potential for reverse causality.

6.4 HANDLING MISSING DATA

We suggest using complete case analysis for models 1, 2, and 3 (i.e. only include participants will all variables). If you decide to handle missing data using a different method for any or all models, please describe below.

**Notes/Comments on Handling Missing Data**

Please describe how missing data was addressed. Include % of data missing for each variable and your method for handling missing data or any additional sensitivity analyses.

7 Table Shells for Descriptive Characteristics

Instructions:

Please complete the following tables summarizing your study's descriptive characteristics. You can fill in the tables yourself or you can provide us with the statistical output and we will enter the values. When providing statistical output, please try to follow this table order and label/title your output with the table # and title.

Please do not hesitate to contact the study manager, Amanda Paluch at apaluch@umass.edu and/or the lead analyst, Carl Pieper at carl.pieper@duke.edu. We are here to help!

7.1 Cohort-level Summary Characteristics

Table 1. Study Description

		(Y/N) or Comment
STUDY SUMMARY	Name of study	
	Contact person	
	Year(s) at baseline data collection (first time point for device measurement)	
	Years(s) at follow-up data collection (last time point for follow-up)	
	Length of follow-up time (mean ± SD)	

Table 1 Continued. Study Description. Please complete the applicable fields for your study.

		**(Y/N) or Comment**
DEVICE PROTOCOL	Type of device and brand	
	Device settings (i.e epoch length)	
	% (n) of total sample that measured steps<br>(total sample = sample at the specific time point when devices were employed)	
	Device wear instructions<br>(i.e. how many total days instructed to wear, 24 hours or waking hours only, and/or no water activities, wear location on body)	
	Corrections or calibrations used	
	Criteria for omission of outliers/truncation of steps data	
	Did you perform any imputation of steps data?	
	If yes, what is the percent (%) of daily step data imputed?	
OUTCOME ASCERTAINMENT	How was Death ascertained?	
	# of deaths	
	How was CVD ascertained or defined?	
	# of fatal CVD events	
	# of non-fatal CVD events	
	# of total (fatal or non-fatal) CVD events	
	How was Type 2 Diabetes ascertained or defined?	
	# Type 2 diabetes events	

7.2 Baseline Descriptive Characteristics

Instructions: Please fill out the descriptive characteristics for ALL participants who meet inclusion criteria. If a variable is not available or quantified in a way other than specified, please add a note/comment in the section below the table.

Table 1A. Descriptive Characteristics at Baseline for Total Sample and by Sex

Variable	Total (n=)		Men (n=)		Women (n=)	
	N or Mean	% or SD	N or Mean	% or SD	N or Mean	% or SD
Age (years), mean(SD)						
BMI (kg/m^2) , mean(SD)						
Race/ethnicity, n (%)						
White						
Black						
Asian						
Hispanic						
Other						
Education variable (categorical, n(%), OR continuous, mean (s.d.)						
BMI Categories, n (%)						
Normal weight (18.5-24.9 kg/m^2)						
Overweight (25.0-29.9 kg/m^2)						
Obese (≥30.0 kg/m^2)						
Hypertension (Stage 2), n (%)						
High cholesterol, n (%)						
Diabetes, n (%)						
History of Cardiovascular disease (%)						
History of Cancer (%)						

*or other SES variable if education is not applicable

For categorical variables, please report **n and %**
For continuous variable, please report the **mean and standard deviation**

How you defined the chronic diseases will be specific to your study.

Complete the table below:

Table 1Aa. Chronic Disease Definitions used in Table 1A

Chronic disease at baseline	Study specific definition/criteria at baseline
Stage 2 Hypertension	
Diabetes	
High Cholesterol	
Cardiovascular Disease	
Cancer	

Table 1B. Additional Descriptive Characteristics at Baseline for Total Sample and by Sex included in your full adjusted MODEL 4*

**Variable**	**Total (n=)**		**Men (n=)**		**Women (n=)**	
	**N or Mean**	**% or SD**	**N or Mean**	**% or SD**	**N or Mean**	**% or SD**

***** Additional demographic, lifestyle, and health status variables included in the full adjusted model (MODEL 4) specific to your study (these may include variables related to: data collection site, smoking status, alcohol, diet, blood pressure, cholesterol, diabetes, CVD, cancer, self-rated health/functional status)
For categorical variables, please report **n and %**
For continuous variable, please report the **mean and standard deviation**

Table 1C. Step Variables at Baseline

	Total (n=)			Men (n=)			Women (n=)		
	**Mean ± S.D.**	**Median [IQR]**	**Range [Min,Max]**	**Mean ± S.D.**	**Median [IQR]**	**Range [Min,Max]**	**Mean ± S.D.**	**Median [IQR]**	**Range [Min,Max]**
**Average steps/day**									
Total Sample									
Quartile 1									
Quartile 2									
Quartile 3									
Quartile 4									
**Cadence Variables**[a]									
Peak 30-min cadence (steps/min)									
Peak 60-min cadence (steps/min)									
Time spent in stepping rate of 0 steps/min:									
Average minutes/day									
Average % of daily wear time									
Time spent in stepping rate of 1-39 steps/min									
Average mins/day									
Average % of daily wear time									
Time spent in stepping rate of 40-100 steps/min:									
Average mins/day									
Average % of daily wear time									
Time spent in stepping rate of ≥40 steps/min:									
Average mins/day									
Average % of daily wear time									
Time spent in stepping rate of ≥100 steps/min:									
Average mins/day									
Average % of daily wear time									
**Compliance Variables**[b]									
No. of days with compliant wear (days)									
Minutes/day of wear on compliant days									

Abbreviations: min(s); minute(s)
[a]Cadence variables will not be available for some studies with pedometer data.
[b]See section 4 for additional details on compliance settings

Notes/Comments on Descriptive Characteristics Tables 1A-1C

Figure 1. Histogram of Distribution of Average Steps/Day per Individual at Baseline

[Insert Histogram here]

7.3 RESULT TABLES – ALL-CAUSE MORTALITY

Instructions: Please complete the analyses with the outcome of All-Cause Mortality using the statistical analysis plan outlined in the document labeled "Steps and Health Meta-Analysis - Statistical Analyses Instructions and Descriptive Table Shells".

You can fill in the tables yourself or you can provide us with the statistical output and we will enter the values. When providing statistical output, please try to follow this table order and label/title your output with the table # and title.

Please do not hesitate to contact the study manager, Amanda Paluch at apaluch@umass.edu, and/or the lead analyst, Carl Pieper at carl.pieper@duke.edu. We are here to help!

Table 2. Unadjusted Event Rate of All-Cause Mortality

	**Steps/day Median [IQR]**	**Steps/day Mean (S.D.)**	**n Events/n Total (and %)**	**Person years of follow-up**	**Cases per 10,000 person-years**
Total Sample					
Categorical					
Quartile 1 – least active					
Quartile 2					
Quartile 3					
Quartile 4 – most active					

Table 3: Hazard Ratios of the Association of Steps/day and All-Cause Mortality

	Model 1 HR (95% CI)	Model 2 HR (95% CI)	Model 3 HR (95% CI)	Model 4 HR (95% CI)
Continuous (per 1,000 steps/day)				
Categorical				
Quartile 1 – least active (ref)				
Quartile 2				
Quartile 3				
Quartile 4 – most active				
p-value for trend				

Model 1: Adjust models for age and sex (if applicable)
Model 2: Model 1 + race/ethnicity, SES, and BMI (as continuous variable)
Model 3: Model 2 + device wear time (if applicable; minutes/day),
Model 4: Model 3 + any additional covariates specific to your study*

Model 3 variables (please list all included variable below):

Table 4. Hazard Ratios for the Association of All-Cause Mortality by Daily Stepping Intensity/Cadence

	Mean (S.D) of Cadence Variable	Median [IQR] of Cadence Variable	n Events/n Total (and %)	Model 1 HR (95% CI)	Model 4 HR (95% CI)	Model 5 (Residual Method adjusted for Steps/day)	
						n Events/n Total (and %)	HR (95%CI)
Peak 30 min Cadence							
Continuous (per 20 steps/min faster)							
Quartile 1 – lowest (ref)							
Quartile 2							
Quartile 3							
Quartile 4 – highest cadence							
p-value for trend							
Peak 60 min Cadence							
Continuous (per 20 steps/min)							
Quartile 1 – lowest (ref)							
Quartile 2							
Quartile 3							
Quartile 4 – highest cadence							
p-value for trend							
Time Spent in Stepping Rate of ≥40 Steps/min							
Continuous (per 10 min/day greater)							
Quartile 1 – lowest min/day (ref)							
Quartile 2							
Quartile 3							
Quartile 4 – highest min/day spent at ≥40							
p-value for trend							
Time Spent in Stepping Rate of ≥100 Steps/min							
Continuous (per 10 min/day)							
Quartile 1 – lowest min/day (ref)							
Quartile 2							
Quartile 3							
Quartile 4							
p-value for trend							

Model 1: Adjust models for age, sex (if applicable)
Model 4: Model 1 + race/ethnicity, SES, BMI, and average wear time and any additional variables specific for your study
Model 5: Model 4 + <u>**steps/day using the residual method**</u>

Model 3 variables (please list all included variables below):

**Notes/Comments on Primary Results Tables/Figures**
Below please describe any unique variables or differences in quantification methods for any variables

7.3.1 Stratification and Sensitivity Analyses

Complete Supplemental Tables (Tables 5A-5D) on the Association of All-Cause Mortality by Steps/Day with the following stratifications:

Table 5A. Sex (male/female)
Table 5B. Age (<60, >=60 years at baseline)

Complete Supplemental Table on the Association of All-Cause Mortality by Steps/Day excluding:

Table 5D. Individuals who died within the first 2 years of follow-up

Supplement Table 5A. Dose Response Association of Steps/Day with All-Cause Mortality by Sex

	Steps/day Median [IQR]	Unadjusted Event Rate			Adjusted Hazard Ratio	
		n Events/ n Total (and %)	Person years of follow-up	Cases per 10,000 person-years	Model 1 HR (95% CI)	Model 4 HR (95% CI)
MEN ONLY						
Continuous (per 1,000 steps/day)						
Categorical						
Quartile 1 – least active (ref)						
Quartile 2						
Quartile 3						
Quartile 4 – most active						
p-value for trend						
WOMEN ONLY						
Continuous (per 1,000 steps/day)						
Categorical						
Quartile 1 – least active (ref)						
Quartile 2						
Quartile 3						
Quartile 4 – most active						
p-value for trend						

Model 1: Adjust models for age, sex (if applicable)

Model 4: Adjust models for age, sex (if applicable), average wear time (if applicable; minutes/day), race/ethnicity, SES, BMI + any additional study specific variables

For the 'total' Hazard Ratios as a continuous variable – quantify as per 1000 steps/day greater

Supplement Table 5B. Dose Response Association of Steps/Day with All-Cause Mortality by AGE

	Steps/day Median [IQR]	Unadjusted Event Rate			Adjusted Hazard Ratio	
		n Events/n Total (%)	Person years of follow-up	Cases per 10,000 person-years	Model 1 HR (95% CI)	Model 4 HR (95% CI)
Young-Middle Age Adults < 60 years old						
Continuous (per 1,000 steps/day)						
Categorical						
Quartile 1 – least active (ref)						
Quartile 2						
Quartile 3						
Quartile 4 – most active						
p-value for trend						
Older Adults ≥ 60 years old						
Continuous (per 1,000 steps/day)						
Categorical						
Quartile 1 – least active (ref)						
Quartile 2						
Quartile 3						
Quartile 4 – most active						
p-value for trend						

Model 1: Adjust models for age, sex (if applicable)

Model 4: Adjust models for age, sex (if applicable), average wear time (if applicable; minutes/day), race/ethnicity, SES, BMI + any additional study specific variables

For the 'total' Hazard Ratios as a continuous variable – quantify as per 1000 steps/day greater

Supplement Table 5C. Dose Response Association of Steps/Day with All-Cause Mortality Excluding Individuals who Died in first 2 Years*

	Steps/day Median [IQR]	Unadjusted Event Rates			Adjusted Hazard Ratio	
		n Events/n Total (and %)	Person years of follow-up	Cases per 10,000 person-years	Model 1 HR (95% CI)	Model 4 HR (95% CI)
Total Sample Continuous (per 1,000 steps/day)						
Categorical						
Quartile 1 – least active (ref)						
Quartile 2						
Quartile 3						
Quartile 4 – most active						
p-trend						

Model 1: Adjust models for age, sex (if applicable)

Model 4: Adjust models for age, sex (if applicable), average wear time (if applicable; minutes/day), race/ethnicity, SES, BMI + any additional demographic, lifestyle, and health status variables to include for a fully adjusted model specific to your study (these may include variables related to: data collection site, smoking status, alcohol, diet, hypertension, high cholesterol, diabetes, CVD, cancer, self-rated health/functional status

For the 'total' Hazard Ratios as a continuous variable – quantify as per 1000 steps/day greater

excluding individuals who died in first 2 years of follow-up
